# Fluorescence-Based Enzyme Activity Assay: Ascertaining the Activity and Inhibition of Endocannabinoid Hydrolytic Enzymes

**DOI:** 10.3390/ijms25147693

**Published:** 2024-07-13

**Authors:** Pierangela Ciuffreda, Ornella Xynomilakis, Silvana Casati, Roberta Ottria

**Affiliations:** Dipartimento di Scienze Biomediche e Cliniche, Università degli Studi di Milano, 20157 Milan, Italy; pierangela.ciuffreda@unimi.it (P.C.); ornella.xynomilakis@unimi.it (O.X.); silvana.casati@unimi.it (S.C.)

**Keywords:** fluorescent assay, endocannabinoid hydrolytic enzymes, high-throughput screening, monoacylglycerol lipase, fatty acid amide hydrolase, *N*-acylethanolamine-hydrolyzing acid amidase

## Abstract

The endocannabinoid system, known for its regulatory role in various physiological processes, relies on the activities of several hydrolytic enzymes, such as fatty acid amide hydrolase (FAAH), *N*-acylethanolamine-hydrolyzing acid amidase (NAAA), monoacylglycerol lipase (MAGL), and α/β-hydrolase domains 6 (ABHD6) and 12 (ABHD12), to maintain homeostasis. Accurate measurement of these enzymes’ activities is crucial for understanding their function and for the development of potential therapeutic agents. Fluorometric assays, which offer high sensitivity, specificity, and real-time monitoring capabilities, have become essential tools in enzymatic studies. This review provides a comprehensive overview of the principles behind these assays, the various substrates and fluorophores used, and advances in assay techniques used not only for the determination of the kinetic mechanisms of enzyme reactions but also for setting up kinetic assays for the high-throughput screening of each critical enzyme involved in endocannabinoid degradation. Through this comprehensive review, we aim to highlight the strengths and limitations of current fluorometric assays and suggest future directions for improving the measurement of enzyme activity in the endocannabinoid system.

## 1. Introduction

Endocannabinoids (ECs) are a class of lipid signaling molecules, isolated from brain and peripheral tissues, that structurally can be described as the amides, esters, and ethers of long-chain polyunsaturated fatty acids. Anandamide (*N*-arachidonoylethanolamine; AEA) and 2-arachidonoylglycerol (2-AG), the first ECs discovered and, consequently, the best studied, are the main endogenous agonists of cannabinoid receptors (CBs) and mimic the pharmacological effects of Δ-9-tetrahydrocannabinol. Both AEA and 2-AG are often found together, but their individual levels vary between species, tissues, developmental stages, and pathophysiological conditions [1]. In addition to AEA, ethanolamides of various long-chain fatty acids, *N*-acylethanolamines (NAEs; i.e., *N*-palmitoylethanolamine (PEA), *N*-oleoylethanolamine (OEA), and oleamide (OA)), are also present in the body. NAEs with derivatives of saturated and monounsaturated fatty acids are much more abundant than anandamide. NAEs, apart from AEA, have no or very low affinity for cannabinoid receptors, and their biological activity is mediated by G protein receptors (GPRs) such as GPR12, GRP10, GPR119, and GPR55, ion channels such as Transient Receptor Potential Vanilloid 1 (TPRV1), or nuclear receptors such as Peroxisome Proliferator-Activated Receptors (PPARs) [2]. The roles of ECs and NAEs in physiological and pathological processes have been extensively studied, revealing the expression of the endocannabinoid system (ECS) quite ubiquitously in the body [3]. As examples, ECs and cannabinoids are involved in metabolism and nutrition [4,5] the gut microbiome [6], and neuropathic, chronic [7], pancreatic [8,9], and orthopedic [10,11,12,13] pain.

ECs and NAEs are produced as a consequence of different biological stimuli, as intracellular calcium increases, and are released from neurons to act paracrinally or autocrinally [14]. These signaling molecules, indeed, cannot be stored in intracellular vesicles and released as classical neurotransmitters and neuropeptides due to their ability to cross cell membranes. Following their release, the signaling function of the ECs is terminated by a two-step inactivation process that consists of uptake mediated by a specific reuptake protein [15] and subsequent intracellular hydrolysis to free the fatty acids and ethanolamine or glycerol by either fatty acid amide hydrolase (FAAH) [16] or monoacylglycerol lipase (MAGL) [17] or by other enzymes, such as *N*-acylethanolamine-hydrolyzing acid amidase (NAAA) or α/β-hydrolase domains 6 and 12 (ABHD6 and ABHD12), in vivo. The association of the ECS and its dysregulation with physiological and pathological processes prompted researchers to study more and more sensible methods to assess ECs and NAEs [18,19,20,21] based on the availability of pure standards [22,23]. On the other hand, the in-depth study of EC and NAE metabolisms paved the way to promote the main components of the ECS as pharmacological targets [24,25]. The enzymes involved in EC metabolism, indeed, are emerging as very important regulators of ECS activity at peripheral and neuronal levels. Synthetic and degradative enzymes of ECs and NAEs offer intriguing opportunities for targeted drug development, considering the numerous biological functions of these lipid signaling molecules in animal tissues [26,27,28,29]. The manipulation of endogenous cannabinoid signaling through the inhibition of hydrolytic enzymes is an attractive approach to elicit the desirable effects of ECS activation while avoiding the negative effects of global EC receptor stimulation. The inhibition of these enzymes would be expected to elevate the endogenous concentrations of all its substrates and, consequently, prolong and potentiate their biological effects. While the roles of enzymes involved in EC and NAE biosynthesis, such as *N*-acyltransacylase (NAT) and *N*-acylphosphatidylethanolamide-phospholipase D (NAPE-PLD), or membrane transport, where anandamide membrane transporter (AMT) seems to be clear, the roles of enzymes involved in their degradation, as FAAH, NAAA, and MAGL, and in controlling EC and NAE cellular activities seem more critical. Otherwise, evidence of the therapeutic potential of EC hydrolytic enzyme inhibitors in combating a variety of human diseases through the bidirectional manipulation of ECs, eicosanoids, other eicosanoid pathways, and other lipid signaling pathways has also been extensively produced [30,31,32,33,34]. The measurement of hydrolytic enzyme activity in physio-pathological conditions will largely contribute both to develop selective inhibitors, which are useful for the creation of novel therapeutic drugs, and to better understand their involvement in pathophysiological processes. With this intent, numerous probes and methods based on different techniques comprising radio tracing [35,36], immunohistochemistry [37,38], fluorescence [39,40,41], and mass analysis [42] have been developed to explore the possibility of studying enzyme localization or activity.

The significant interest of the scientific community in this field has resulted in numerous publications predominantly concentrating on enzyme localization. This focus has made it challenging to readily identify high-throughput screening (HTS) or activity assay methods that are useful for drug development. For this reason, this review will provide a detailed overview of the current knowledge regarding the monitoring of enzyme activities involved in EC degradation using fluorometric methods. The methods and probes used for determining the kinetic mechanisms of enzyme reactions, as well as for configuring kinetic assays for HTS, have been considered.

## 2. Enzymes Involved in Endocannabinoid Hydrolysis

AEA is mainly hydrolyzed by FAAH, while 2-AG is mainly hydrolyzed by MAGL and FAAH. In addition to these two principal enzymes, NAAA and, more recently, a second FAAH (FAAH-2) as well as two other serine hydrolases, ABHD6 and ABHD12 [43], have been reported to participate in the degradation of several ECs.

### 2.1. MAGL

**Figure 1:**
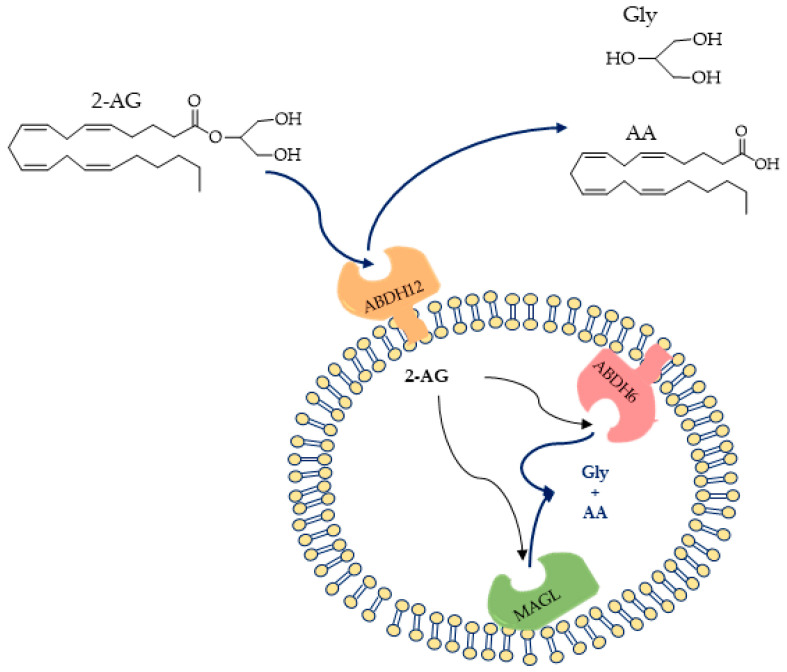
(Up) Structures of 2-arachidonoylglycerol (2-AG) and the hydrolysis products; (down) proposed orientations of 2-AG hydrolysis enzymes (ABHD6, ABHD12, and MAGL) [50].

Monoacilglycerol lipase (MAGL EC3.1.1.23, acylglycerol lipase), belongs to the α/β-hydrolase domain (ABHD) family of serine hydrolases, containing a classical Ser-His-Asp catalytic triad [44,45]. It is a soluble enzyme associated with membranes [17,46]. MAGL catalyzes a reaction that uses water molecules to break down 2-AG into glycerol and arachidonic acid [47]. The enzyme is found both in the brain and in peripheral tissues such as the kidney, ovary, testis, adrenal gland, adipose tissue, and heart [47], where it preferentially hydrolyzes monoacylglycerols to glycerol and the corresponding fatty acid, in particular the endocannabinoid 2-AG [48]. It has been demonstrated by pharmacological inactivation or genetic silencing that MAGL is responsible for 80% of 2-AG hydrolysis in many tissues, including the brain. The remaining 20% of hydrolysis of 2-AG is due to the serine hydrolases ABHD6 and ABHD12 (Figure 1) [43,49].

Several studies measuring the rate of hydrolysis report the impact of the length and degree of unsaturation of the substrate acyl chain. MAGL efficiently hydrolyzes substrates with various acyl chain lengths (from C8:0 to C18:0) and different numbers of double bonds (C18:1, C18:2; and C20:4). The degree of unsaturation was reported to have a certain impact, with a preference for arachidonoylglycerol compared to palmitoylglycerol and, in general, for unsaturated compared to saturated substrates [51]. Consequently, the selective inactivation of MAGL could be a useful pharmacological tool for different pathological conditions due to its major role in 2-AG degradation. ECs, even 2-AG, are involved in inflammation, as they are physiologically produced in response to injury with the aim of decreasing pro-inflammatory mediators. Elevated levels of 2-AG have been observed in various neuropathologies characterized by inflammatory involvement, including multiple sclerosis, Parkinson’s disease, and Alzheimer’s disease.

Moreover, inhibiting MAGL not only promotes an increase in 2-AG, which is beneficial for its anti-inflammatory function, but also induces a reduction in free arachidonic acid (AA) levels, thereby preventing the activation of the eicosanoid pathway [52]. In addition, in 2010, by controlling fatty acid release from lipid stores, Nomura and coworkers revealed the involvement of MAGL in cancer cell migration, proliferation, and tumor growth as a precursor of tumorigenic lipid messengers. MAGL is indeed up-regulated in cancers such as ovarian, breast, and melanoma, and its inhibition leads to reduced cell invasiveness and tumorigenicity [53].

### 2.2. ABHD6 and ABHD12

The α/β-hydrolase enzymes belong to a family of newly discovered enzymes that degrade ECs. The first evidence that MAGL hydrolyzes 2-AG was provided by Muccioli et al. in the mouse microglial cell line BV-2, which does not express MAGL mRNA but efficiently hydrolyzes 2-AG [54]. While MAGL has been structurally and functionally analyzed, ABHD6 and ABHD12 remain poorly characterized at the molecular level. Both are membrane proteins, and it is postulated that the catalytic domain of ABHD6 faces the cytoplasm, whereas ABHD12 seems to be a transmembrane glycoprotein with its active site facing the extracellular space [55].

#### 2.2.1. ABHD6

ABHD6, an integral membrane protein that is ubiquitously expressed [56,57], is a serine hydrolase with 337 amino acids (38 kDa) with a catalytic triad composed of Ser148-sp278-His306. In vitro experiments demonstrated that ABHD6, like MAGL, is much more active on 2-monoglycerides comprising medium to long and saturated acyl chains. These two enzymes can therefore handle different MAGs due to their availability in the cell cytoplasm [58]. ABHD6 is an important enzyme involved in many physiological and pathological states, not only in the central nervous system but also in peripheral tissues [50,59]. Accordingly, ABHD6 has been implicated in the modulation of various (patho)physiological processes, including metabolic syndrome [57], inflammation [60], insulin secretion [61], obesity [62], adipose browning and brown adipose activation [62], cancer [63], and neurological diseases, making it a promising therapeutic target to treat several diseases [50].

#### 2.2.2. ABHD12

ABHD12, known as ABHD12 or 2-arachidonoylglycerol hydrolase, is a single-pass integral membrane glycoprotein of 398 residues (45 kDa). ABHD12 is ubiquitously expressed in the body but has the highest expression in the brain [64], where it is responsible for about 9% of 2-AG hydrolysis [43]. To date, 2-AG is the only recognized substrate for ABHD12, even if studies performing a fluorescent glycerol assay demonstrated ABHD12’s preference for the 1 (3)-monoglycerides of arachidonic acid and polyunsaturated chains in respect to saturated (C20:4 > C18:2 > C14:0) [58]. Moreover, ABHD12, which has an active site facing the extracellular space, has been proposed as a suitable guardian of the extracellular signaling pool of 2-AG.

### 2.3. FAAH

**Figure 2:**
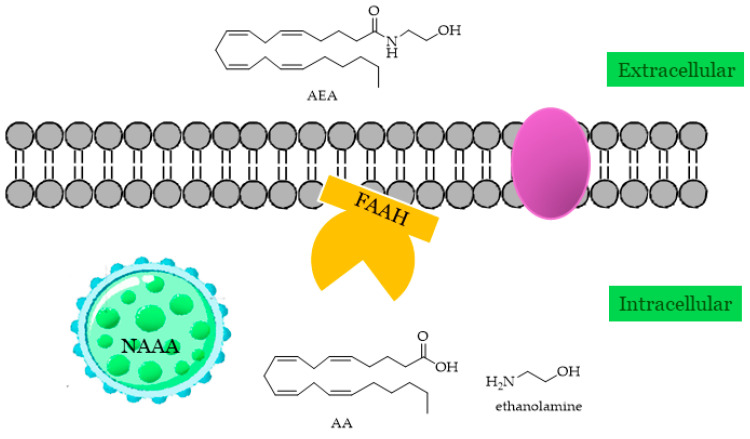
Localizations of AEA-hydrolyzing enzymes (FAAH and NAAA).

Fatty acid amide hydrolase (FAAH, oleamide hydrolase, anandamide amidohydrolase; EC 3.5.1.99) is the main actor in the degradation of AEA and NAEs including PEA, OEA, the sleep-inducing lipid OA [16], and N-acyl taurines (NATs), agonists of the transient receptor potential (TRP) family of calcium channels [65]. FAAH not only hydrolyzes AEA to arachidonic acid and ethanolamine but also 2-AG, and it is widely distributed throughout the body and has been localized in the intracellular space associated with the membrane (Figure 2). Human FAAH mRNA has been found in the pancreas, kidney, brain, and skeletal muscles, while smaller amounts have been found in the placenta and liver.

FAAH is a membrane-bound enzyme that belongs to the family of amidase proteins. FAAH’s active site possesses an unusual serine–serine–lysine (Ser217-Ser241-Lys142) catalytic triad that is characteristic of the amidase signature class of enzymes. This “amidase signature” region is common to more than 100 amidases, most of which are bacterial and fungal in origin [66], and is different from the typical Ser-His-Asp catalytic triad utilized by most of the other serine hydrolases. FAAH appears to play a major role in regulating the amplitude and duration of fatty acid amide signals in vivo [67], suggesting its potential as a significant modulating enzyme for a number of neurobehavioral processes in mammals, including pain, sleep, feeding, and locomotor activity. An FAAH activity blockade also leads to very high endogenous levels of fatty acid amides in the nervous system [68] and peripheral tissues [69], resulting in analgesic [68], anxiolytic [68], and anti-inflammatory [69] effects without showing the undesirable side effects linked to direct cannabinoid receptor agonists on motility, cognition, or body temperature [68,70]. These findings suggest that FAAH may represent an attractive therapeutic target for the treatment of pain, inflammation, and numerous central nervous system (CNS) disorders.

### 2.4. NAAA

The primary route for breaking down NAEs into free fatty acids and ethanolamine involves their hydrolysis, which has been mostly attributed to FAAH. However, an additional enzyme found in lysosomes, referred to NAAA, has demonstrated capability in catalyzing the same reactions (Figure 2). NAAA, a lysosomal glycoprotein abundantly expressed in macrophages, is not a member of the serine hydrolase family; instead, it operates as an *N*-terminal nucleophilic hydrolase (Ntn), specifically as an N-terminal cysteine hydrolase within the choloylglycine hydrolase family, which is characterized by the ability to cleave non-peptide amides [71,72]. Despite its functional similarity with FAAH, NAAA shares no homology with this enzyme, but it is much more similar to acid ceramidase (AC), the lysosomal enzyme responsible for the hydrolysis of ceramide to sphingosine and free fatty acid, showing 33–34% identity similarity with it. Consistent with its lysosomal localization, NAAA is active at an acidic pH (pH 4.5), a condition necessary for its conversion to the shorter active form by proteolysis from the zymogen [71,73]. NAAA exhibits its highest activity towards PEA [73], an important regulator of energy balance, pain, and inflammation, and was recently suggested as a contributor to the control of reward-related behaviors. Since the actions of PEA are terminated via enzyme-mediated hydrolysis, which is catalyzed by NAAA, this enzyme could be a promising therapeutic target [74,75], for example, in modulating inflammatory responses. However, unlike FAAH and MAGL, there is a notable absence of direct structural information available on this enzyme.

## 3. Fluorescence-Based Assay for Biological Applications

In recent years, enzyme-activated fluorescent probe-based assays are probably the most widely used detection method in drug screening and disease diagnosis due to their capability of being applicable for HTS experiments. This widespread use is largely due to their high sensitivity, good tolerance to interference, fast signaling speed, high versatility, and simplicity. Moreover, the non-destructive way of tracking or analyzing targets and diverse selection of fluorophores that excite and emit across a broad spectrum of wavelengths make them the first choice in enzymology research [76,77].

Fluorescence-based detection techniques enable miniaturization, flexible assay design, ease of use, and simultaneous monitoring of multiple events. These assays are divided into two types based on the fluorescent signals collected: those detecting total fluorescence intensity [78], polarization [79], resonance energy transfer [80], lifetime [81], and time-resolved fluorescence [82] and those detecting fluorescence from single molecules, like fluorescence correlation spectroscopy [83] and intensity distribution analysis [84]. Enzyme-activated fluorescent probes are widely used to characterize bioactive enzymes due to their high sensitivity, non-invasive monitoring, and real-time sampling capabilities [85,86,87]. This technique has also been extensively used for enzyme activity measurement for the advantages of simplicity, selectivity/sensitivity, and noninvasive detection when used for cell imaging [76,88,89,90].

**Figure 3:**
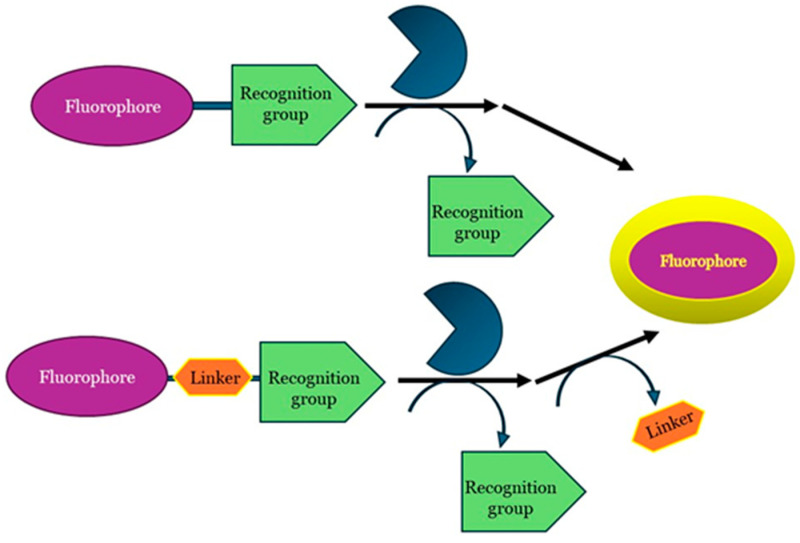
General structure and mechanism. (Up) Substrate-based probes; (down) substrate-based probes containing self-immolate linker.

Fluorescence-based assays require reactions that either lead to the formation of a fluorescent product from a non-fluorescent substrate or vice versa. Fluorescent probes are usually composed of two or three components (Figure 3): (i) a signal or fluorophore moiety; (ii) a recognition or labeling moiety; and (iii) an appropriate linker to connect the two moieties. The recognition motifs (tag) structurally resemble the enzyme substrates in order to drag the probe to the active site of the enzyme and are non- or weakly fluorescent until some event occurs, such as enzymatic cleavage.

For these studies, the design of the probe is a key step of the process. Not only does the recognition moiety have to be like the enzyme substrate to reach its active site, but also the bond between it and the fluorescent dye must be efficiently cleaved by the enzyme. Moreover, a careful selection of the fluorescent molecule is important too due to the correct balance of two fundamental requirements: a good affinity with the enzyme and a fast release. Potential fluorophores could indeed perfectly fit with the catalytic site of the enzyme but not lead to the release of the fluorescent dye. Fluorescence enzyme assays are generally more sensitive than spectrophotometric assays but can be affected by impurities and the instability of fluorescent compounds in light, requiring careful handling. They can also be designed in various formats based on experimental needs.

## 4. Fluorescence-Based Assays Used to Screen and Characterize Crucial Enzymes Involved in EC Hydrolysis

Enzyme assays can be used for a variety of purposes, including the localization of an enzyme to prove its expression in a distinct specimen, like an organism or a tissue, or the investigation of specific enzyme activities and kinetics as well as the study of enzyme modulators. Enzyme activity is the rate of enzyme reaction, generally expressed in units (U), of a substrate converted (or product formed) per time unit per milligram of enzyme under a given set of conditions (substrate, concentration, solvent, buffer, and temperature). According to the currently valid SI system, the concentration should be in mol/L and the time unit in seconds. All the enzymatic in vitro reactions include a substrate that is consumed and converted into a product, whose time-dependent formation is analyzed and quantified. The most popular assays are those that produce a spectrophotometric signal and use simple reagents, in particular chromogenic or fluorogenic substrates. In recent years, fluorimetric techniques have gained significant relevance when compared to UV/Vis spectrophotometry techniques due to the following:

- They are more sensitive;
- Reaction kinetics can be monitored continuously;
- They require a relatively low amount of enzyme, usually between 0.005 and 0.5 mg/mL;
- They require a relatively low amount of substrate, usually between 0.0025 and 0.25 mM;
- They allow for the use of different buffers under different reaction conditions;
- They allow for micro-assay analysis, as they not only utilize a fluorometer (for one sample at a time) but also enable the use of a microplate reader with fluorimetric detection for the quantification of multiple samples simultaneously.

Considering the different applications of fluorescence in the study of the EC hydrolytic enzymes and the extensive literature on the subject, this review is not intended to cover such an enormous scope of fluorescence-based applications. Instead, it will focus on enzyme-activated fluorescent probes for the evaluation of EC and NAE hydrolysis.

However, fluorescence-based assays are not without limitations, which deserve careful consideration. A significant disadvantage of fluorescence-based assays is background interference due to impurities in the samples or the autofluorescence of biomolecules, such as in the case of experiments with cells, which can obscure the signal of interest. This requires meticulous sample preparation and control experiments to distinguish real signals from noise. Achieving accurate quantitative measurements can be challenging in fluorescence assays. Factors such as photobleaching of fluorophores, variations in the excitation light intensity, and non-linear fluorescence responses complicate the calibration and quantification process. Careful standardization and calibration procedures are required to ensure reliable quantitative data. Fluorophores are susceptible to photobleaching, where prolonged exposure to excitation light leads to an irreversible loss of fluorescence signal. This imposes constraints on the assay duration and necessitates careful control of light exposure to maintain signal integrity throughout the experiment. Also, environmental factors such as the pH, temperature, and solvent polarity can influence signals, altering fluorophore properties and affecting assay reproducibility and reliability. Rigorous environmental control and validation procedures are essential to minimize these effects. Fluorescence assays are typically limited in their ability to multiplex the simultaneous measure of multiple analytes in the same sample due to spectral overlap between different fluorophores, which can interfere with signal detection and quantification. Strategies such as the use of different wavelengths or time-resolved fluorescence techniques are used to overcome these limitations.

### 4.1. MAGL

MAGL is the main enzyme responsible for the hydrolysis of 2-AG. The predominant assay methods for screening and characterizing MAGL activity modulators involve measuring the breakdown products of radiolabeled MAGL substrates [17,45] or detecting the release of arachidonic acid resulting from 2-AG hydrolysis using high-performance liquid chromatography (HPLC) coupled with UV [91] or mass spectrometric detection [92]. These methods are highly sensitive and accurate, using, de facto, the endogenous substrate as the probe but, on the other hand, require the use of radioactive labeled compounds and purification and extraction procedures coupled with the use of expensive instrumentation and are not particularly amenable for HTS [17,93,94]. In this scenario, an alternative approach based on the evaluation of the rate of MAGL-catalyzed hydrolysis of p-nitrophenyl alkyl esters by the UV monitoring of the release of p-nitrophenol was proposed by Miccioli in 2008 [95]. Taking advantage of these features, several fluorogenic probes have been finalized for the detection of MAGL, which are based on the different emission properties before and after the deacylation of fluorophores. Several research attempts have focused on developing reliable, straightforward, cost-effective, and sensitive as well as high-throughput assays for investigating the activity of MAGL. Among these, small-molecule substrate probes based on coumarin and resorufin fluorescent dyes have been synthesized and evaluated.

**Figure 4:**
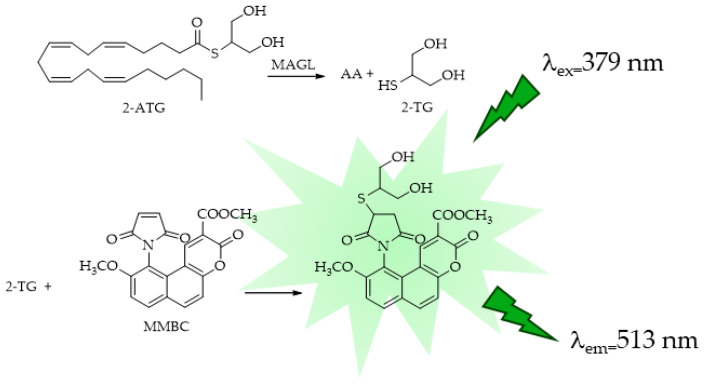
MAGL-responsive fluorescent probe methyl maleimido-benzochromene carboxylate (MMBC).

Starting from the large application of maleimides, which react rapidly and specifically with thiols to give addition products under mild conditions, fluorescent maleimides for the study of protein structure were first designed and synthesized by Kanaoka [96]. This maleimide-based fluorescent probe found wide applications in the study of protein structural and micro-environmental properties, micro-assays for glutathione S-transferase, and as derivatization agent for the HPLC analysis of thiol compounds. Casida et al. [97] reported the synthesis of a thioester analog of the endocannabinoid 2-AG, *S*-arachidonoyl-2-thioglycerol (2-ATG), in an eight- or nine-step procedure with a yield of 25%. This substrate was reacted with methyl maleimido-benzochromenecarboxylate (MMBC) [98] for fluorescent assays of human recombinant MAGL lipase (*h*MAGL) and human brain membrane MAGL hydrolase activity. 2-ATG was found to be an excellent substrate for *h*MAGL and a suitable probe for microplate assays measuring 2-thioglycerol (2-TG) liberation by the reaction with MMBC and the formation of a fluorescent derivative (Figure 4).

**Figure 5:**
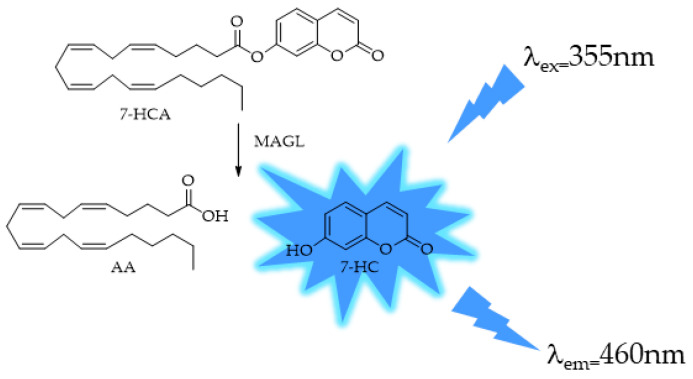
MAGL-responsive fluorescent probe 7-hydroxycoumarinyl-arachidonate (7-HCA).

The method proposed by Casida [97] has seen limited utilization due to the involvement of two enzymes, leading researchers to focus on methods employing a single enzyme. Wang et al. developed a 7-hydroxycoumarinyl-arachidonate (7-HCA) by substituting the glycerol moiety of 2-AG with a fluorescent coumarin derivative [99] (Figure 5).

As shown in Figure 5, MAGL selectively cleaves the ester bond of 7-HCA to generate AA and form the corresponding highly fluorescent 7-hydroxylcoumarin (7-HC). The release of 7-HC is monitored continuously using a fluorimeter. MAGL protein catalyzed the hydrolysis of 7-HCA with an apparent Km of 9.8 mM and Vmax of 1.7 mmoles min^−1^ mg protein^−1^, and the assay was reproducible (*Z*’ 0.7 ÷ 0.9). To reach this method performance, several experiments were performed varying the reaction conditions, pH, DMSO and bovine serum albumin (BSA) concentrations, and the 7-HC auto hydrolysis or the presence of denaturized MAGL. The same method was also applied in a patent (US2009311723A1: Fluorescence-based assay for monoacylglycerol lipase compatible with inhibitor screening) that provides reagents, kits, and methods for assaying MAGL activity and for identifying compounds that modulate MAGL activity. The enzyme used in the patent was a recombinant MAGL, expressed in *E. coli*. The recombinant MAGL can be used in any assay, such as fluorescence MAGL assays and other screens for agents that modulate MAGL activity, and can be included in the mixture at a concentration in the range of 1 ng to 10 ng per 50 μL of total volume of mixture.

**Figure 6:**
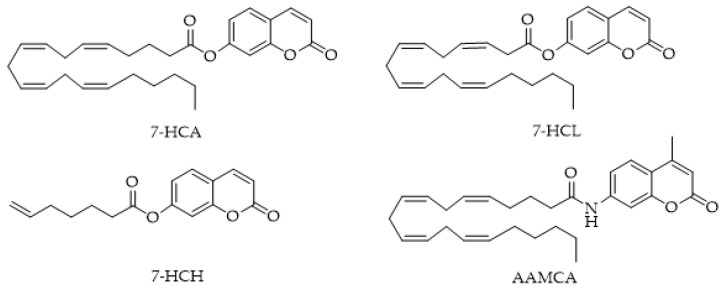
Structures of MAGL-responsive probes 7-hydroxycoumarinyl-arachidonate (7-HCA), 7-hydroxycoumarinyl-γ-linolenate (7-HCL), 7-hydroxycoumarinyl-6-heptenoate (7-HCH), and non-responsive arachidonyl 7-amino-4-methylcoumarin amide (AAMCA).

MAGL also catalyzes the hydrolysis of other ester-linked compounds such as 7-hydroxycoumarinyl-γ-linolenate (7-HCL) and 7-hydroxycoumarinyl-6-heptenoate (7-HCH) (Figure 6). 7-amino-4-methylcoumarin amide (AAMCA), a FAAH substrate [100] that has an amide link, was not a substrate for MAGL.

In 2010, Savinainen et al. [101] improved and further developed the Wang method, a rapid and versatile HTS assay for MAGL inhibitors. Re-examining the original report [99], the highest MAGL activity was detected at pH 9.0–10.0, although the MAGL assays were routinely conducted in a neutral pH range (pH 7–7.4). In addition, it is known that 7-HC is fully deprotonated and, thus, maximally fluorescent at an alkaline pH [102]. Determining the fluorescence of 7-HC over the pH range of 7.4–9.0, the authors observed, as expected, that the fluorescence of 7-HC increased in parallel with an increasing pH. Therefore, they clarified whether the high MAGL activity observed under alkaline conditions reflected this phenomenon. Furthermore, in contrast to conventional MAGL assays, BSA is not compatible with the fluorescent assay using 7-HCA, because BSA dose- and time-dependently enhanced the fluorescence signal even in the absence of MAGL.

**Figure 7:**
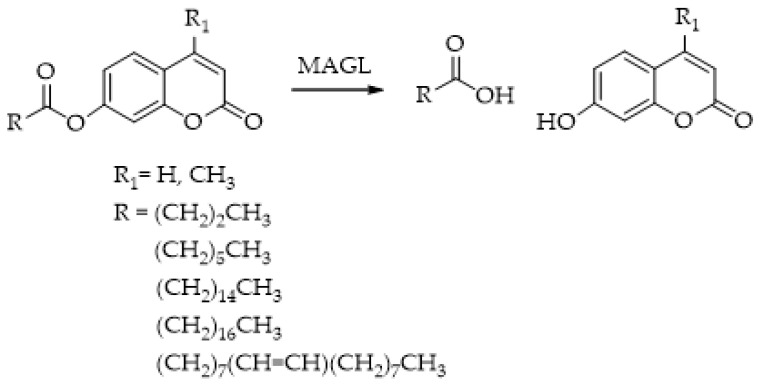
MAGL-responsive fluorescent probe: 4-methylcoumarin and coumarin-based acyl-substrates with various aliphatic chain lengths.

Finally, the authors suggest that instead of using just one single, high concentration of inhibitor in dilution-based experiments to evaluate the reversibility of the inhibition, as is common practice, a wider concentration range is more suitable to completely characterize the behavior of inhibitors. In 2012, the Clemente group [103] reported the results of their study in which five different 4-methylcoumarin and coumarin-based acyl substrates with various aliphatic chain lengths were explored as 2-AG mimics in MAGL activity assays (Figure 7). Before testing enzymatic hydrolysis, the solubility of the substrate was measured, and as expected, the solubility decreased as a function of increasing the aliphatic chain length. The authors demonstrated that MAGL effectively hydrolyzes the proposed coumarin-based substrates with aliphatic chains of varying lengths. Lastly, all subsequent kinetic tests used 4-methylcoumarin butyrate due to its greater solubility.

**Figure 8:**
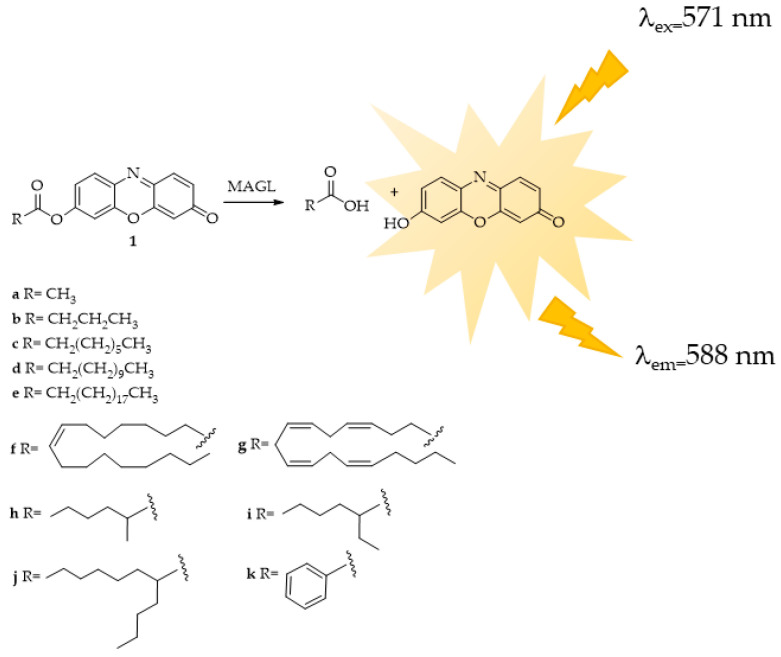
MAGL-responsive fluorescent probes: 7-hydroxyresorufynil esters: acetate (**1a**), butyrate (**1b**), octanoate (**1c**), dodecanoate (**1d**), icosanoate (**1e**), oleate (**1f**), arachidonate (**1g**), 2-methylhexanoate (**1h**), 2-ethylhexanoate (**1i**) 2-butyloctanoate (**1j**), and benzoate (**1k**). For clarity, only the compounds cited directly in the text and figure have been numbered.

The research conducted by Lauria et al. [104] resulted in the successful synthesis of a fluorogenic substrate probe at a long wavelength featuring a resorufin fluorophore (Figure 8, compound **1g**). This probe exhibited significant sensitivity for MAGL, making it applicable for MAGL screening assays and the identification of potential MAGL inhibitors. Subsequently, the same research group [105], with a view to finding a suitable substrate for HTS experiments readily accessible, stable in water solution, and with a low rate of spontaneous hydrolysis, synthesized eleven novel potential substrates. These newly synthesized esters of fluorescent resorufin encompass various classes of acyl chains, including linear chains such as acetate (**1a**), butyrate (**1b**), octanoate (**1c**), dodecanoate (**1d**), icosanoate (**1e**), and oleate (**1f**) as well as branched chains like 2-methylhexanoate (**1h**), 2-ethylhexanoate (**1i**), and 2-butyloctanoate (**1j**) alongside aromatic chains like benzoate (**1k**) (Figure 8). Furthermore, the authors conducted investigations on the substrate specificity of MAGL based on the acyl chain variations, determining the kinetic constants. The differences in substrate interaction with the MAGL active site were also analyzed using structural in-silico techniques.

The best substrate for the HTS method was identified as 7-hydroxyresorufinyl octanoate (**1c**). Among the compounds tested, **1c** exhibited the highest rate of hydrolysis and displayed the most favorable Km and Vmax values. In silico docking studies revealed favorable interactions between **1c** and the MAGL active site. Furthermore, compound **1c** can be easily prepared on both milligram and gram scales and demonstrates high stability in solution, as evidenced by its low rate of spontaneous hydrolysis. The authors validated the proposed probe **1c** using the well-known MAGL inhibitors URB602 and MAFP under the same established assay conditions.

**Figure 9:**
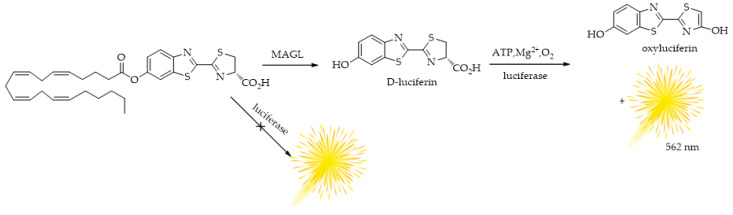
Bioluminescence reactions of the bioluminescence probe for MAGL, arachidonoyl luciferin.

Later, the same research team presented, in 2021 [106], an interesting study on the development of a straightforward assay, taking advantage of the light production from the reaction of luciferase with firefly luciferase. They presented the synthesis of the new bioluminescent probe arachidonoyl-luciferin and the development of a new two-step HTS method for the analysis of MAGL (Figure 9). The method revealed to be a powerful tool for ECS modulation research due to both the selectivity of the probe and the use of an engineered thermostable luciferase (PLG2) with a specific light-emitting enhanced activity (λ_max_ = 559 nm) [107]. The new bioassay for MAGL activity offers significant advantages, including high sensitivity and rapidity, making it suitable for applications where the availability of biological samples is limited.

**Figure 10:**
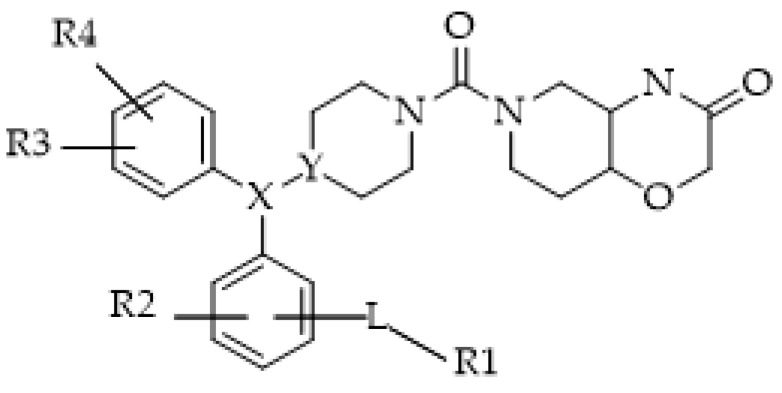
General design of fluorescent probe for MAGL. L is a linker; R1 is a fluorescent label; R2, R3, and R4 are each independently selected from hydrogen, halogen, C1-C6-alkyl, halo-C1-C6-alkyl, C1-C6-alkoxy, and halo-C1-C6-alkoxy; and X and Y are both CH; X and Y taken together form a double bond (C=C).

In 2020, a patent (1. WO2021058443-Fluorescent probes for monoacylglycerol lipase (MAGL)) reported a very wide range of fluorogenic probes for MAGL (Figure 10) mostly used to investigate the localizations, structures, dynamics, and functions of proteins in living cells.

**Figure 11:**
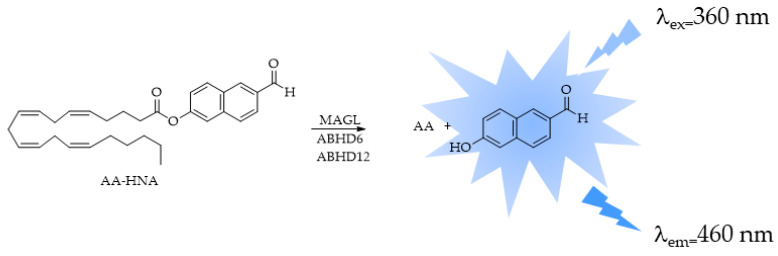
Fluorescence turn-on responses of 6-hydroxy-2-naphthaldehyde-arachidonate (AA-HNA) on 2-AG hydrolases (MAGL, ABHD6, and ABHD12).

Deng et al. [108] developed a new fluorogenic probe for monitoring the activity of MAGL, 6-hydroxy-2-naphthaldehyde-arachidonate (AA-HNA) (Figure 11), and developed an AA-HNA-based fluorescence assay to rapidly identify MAGL inhibitors. This assay was also suitable for the other 2-AG hydrolases, ABHD6 and ABHD12. The assay was used to successfully analyze a focused library containing 320 natural organic compounds.

**Figure 12:**
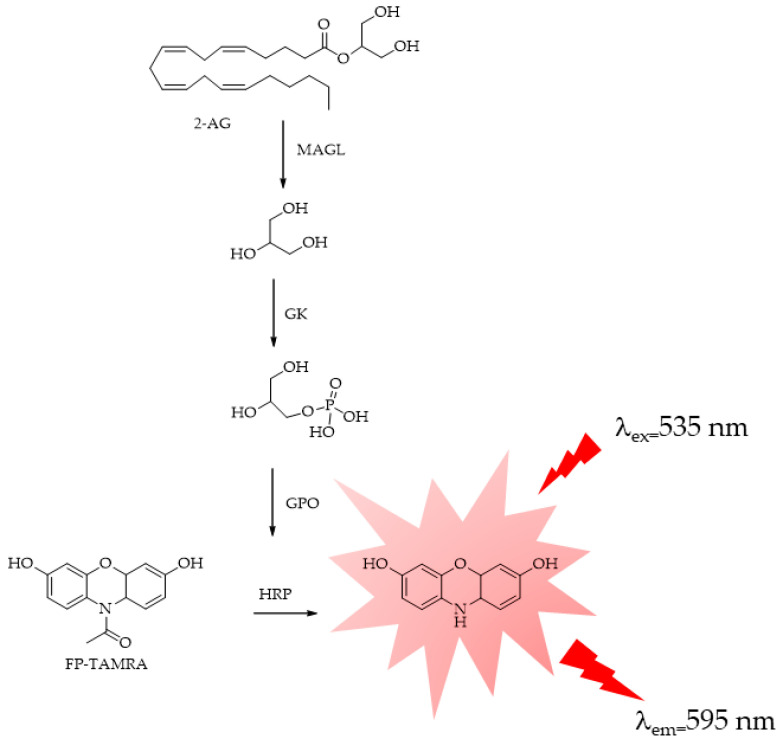
GK = glycerol kinase; GPO = glycerol-3-phosphate.

Recently, Jiang et al. proposed the probe fluorophosphatetramethyl rhodamine (FP-TAMRA), which has large applicability in the detection of serine hydrolases, including MAGL, FAAH, and ABHD6 [109]. FP-TAMRA, by an enzyme-coupled reaction, is able to detect the glycerol-3-phosphate (GPO) produced after 2-AG hydrolysis. The author described the application of the method to assess new MAGL inhibitors (Figure 12).

### 4.2. ABHD6 and ABHD12

**Figure 13:**
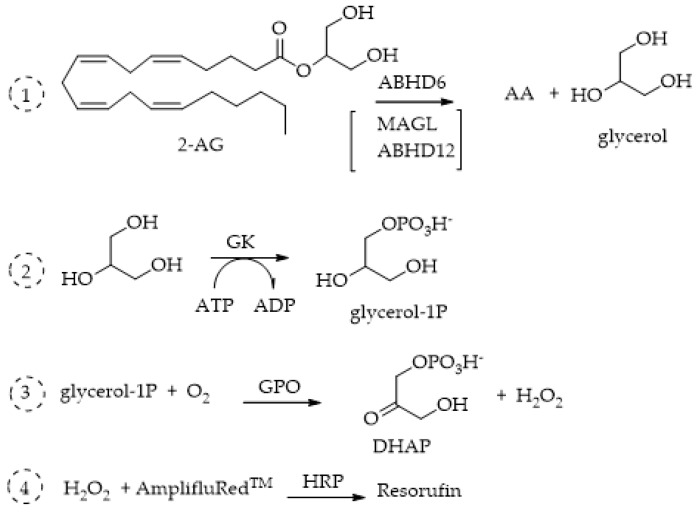
Fluorescence multi-enzymatic method proposed by Savinainen for MAGL, ABHD6, and ABHD12 activity evaluations. 1_ Catalysis of 1(3)-AG hydrolysis by ABHD; 2_ glycerol conversion to glycerol-1-phosphate (G-1-P) in presence of ATP performed by glycerol kinase (GK); 3_ oxidation of G-1-P catalyzed by glycerol 3-phosphate oxidase (GPO), generating H_2_O_2_; 4_ reaction of Amplifu™ Red with H_2_O_2_ catalyzed by horseradish peroxidase (HRP to form fluorescent resorufin.

Few fluorometric methods have been applied to ABHD6 and ABHD12, probably because these enzymes remain even less characterized and studied than MAGL. In 2016, Savinainen’s group reported [110] a sensitive fluorescence-based method for the assessment of ABHD6 activity, which was then validated for the three 2-AG hydrolases, MAGL, ABHD6, and ABHD12, using the human recombinant enzymes produced in HEK293 cells. The proposed method is based on the principle of detecting enzyme activity by coupling a primary enzyme reaction with one or more secondary enzyme reactions to generate a measurable product, in this case resorufin. The operating mechanism of the coupled enzyme system for detecting ABHD6 activity involves the catalysis of 1(3)-AG hydrolysis by ABHD6, resulting in the production of equimolar amounts of AA and glycerol. Within this system, glycerol undergoes conversion to glycerol-1-phosphate (G-1-P) in the presence of ATP, facilitated by glycerol kinase (GK). Subsequently, glycerol 3-phosphate oxidase (GPO) catalyzes the oxidation of G-1-P, generating H_2_O_2_, which, in conjunction with horseradish peroxidase (HRP), transforms Amplifu™ Red (Thermo Fisher Scientific, Waltham, Massachusetts, US) into the fluorescent product resorufin (Figure 13). The fluorescence of resorufin (λ_ex_ 530 nm; λ_em_ 590 nm) is then continuously monitored to track the enzymatic kinetics. The Amplex™ Red Hydrogen Peroxide/Peroxidase Assay kit (Thermo Fisher Scientific, Waltham, Massachusetts, US) features a highly sensitive one-step assay utilizing Amplex™ Red reagent (10-acetyl-3,7-dihydroxyphenoxazine) for the detection of H_2_O_2_ or peroxidase activity. When paired with horseradish peroxidase (HRP), Amplex™ Red reagent enables the identification of H_2_O_2_ released from biological samples such as cells or produced in enzyme-coupled reactions (Figure 13). The technique exhibits remarkable sensitivity, capable of detecting picomolar amounts of glycerol and necessitates merely a small amount of lysate (0.3 μg protein/well) prepared from HEK293 cells transiently expressing ABHD6. The same method has been applied and described for ABHD12 [111].

The only other method for the analysis of ABHD6 and ABHD12 activities employs, as the fluorogenic probe, 7-hydroxy-2-naphthaldehyde-arachidonate (AA-HNA), previously described for MAGL (Figure 11) [108].

### 4.3. FAAH

**Figure 14:**
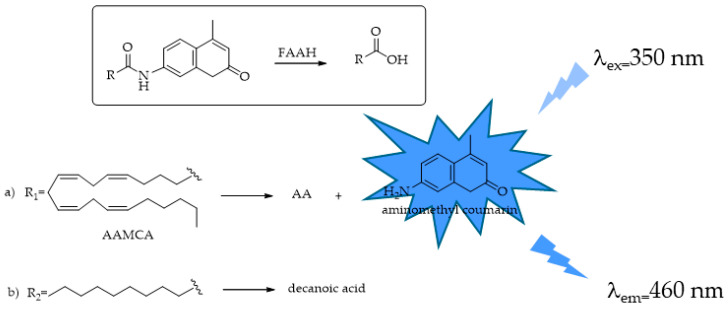
FAAH-responsive fluorescent probe: (**a**) AAMCA = arachidonyl 7-amino, 4-methyl coumarin amide; (**b**) decanoyl aminomethyl coumarin. AA = arachidonic acid.

Fatty acid amide hydrolase (FAAH), a member of the serine hydrolase superfamily, plays a crucial role in the hydrolysis and inactivation of biologically active amides, including endogenous AEA, OEA, and PEA. The traditional assay procedures used to screen and characterize FAAH detection utilize radiolabeled ligands to measure substrate hydrolysis and either TLC or HPLC as the purification step, processes that are both expensive, time consuming [112,113,114,115], and are not particularly amenable for HTS. Other assays monitor the release of AA from AEA by using HPLC coupled with a UV detector [116]. Even in this case, these methods use expensive instrumentation and are not suitable for HTS, requiring lipid extraction and purification. The first colorimetric method proposed for FAAH was based on its ability of hydrolyzing OA to produce ammonia that is then detected and quantified by a second enzymatic process, an NADH/NAD^+^-coupled enzyme reaction [117]. Its application is limited by its sensitivity and the complexity of using a second enzyme (e.g., L-glutamate dehydrogenase). With respect to these methods, fluorogenic probes have drawn considerable attention because of their major simplicity and higher sensitivity. Ramarao et al. [100] proposed the first fluorogenic substrate for FAAH and set up a simple approach for rapid fluorescence detection utilizing arachidonyl 7-amino, 4-methyl coumarin amide (AAMCA). The non-fluorescent AAMCA, AA linked to coumarin by the amide bond, is specifically hydrolyzed by FAAH to produce AA and the highly fluorescent 7- amino, 4-methyl coumarin (AMC) (λ_ex_ = 355 nm, λ_em_ = 460 nm, Figure 14) that can be readily detected using a fluorometer.

**Figure 15:**
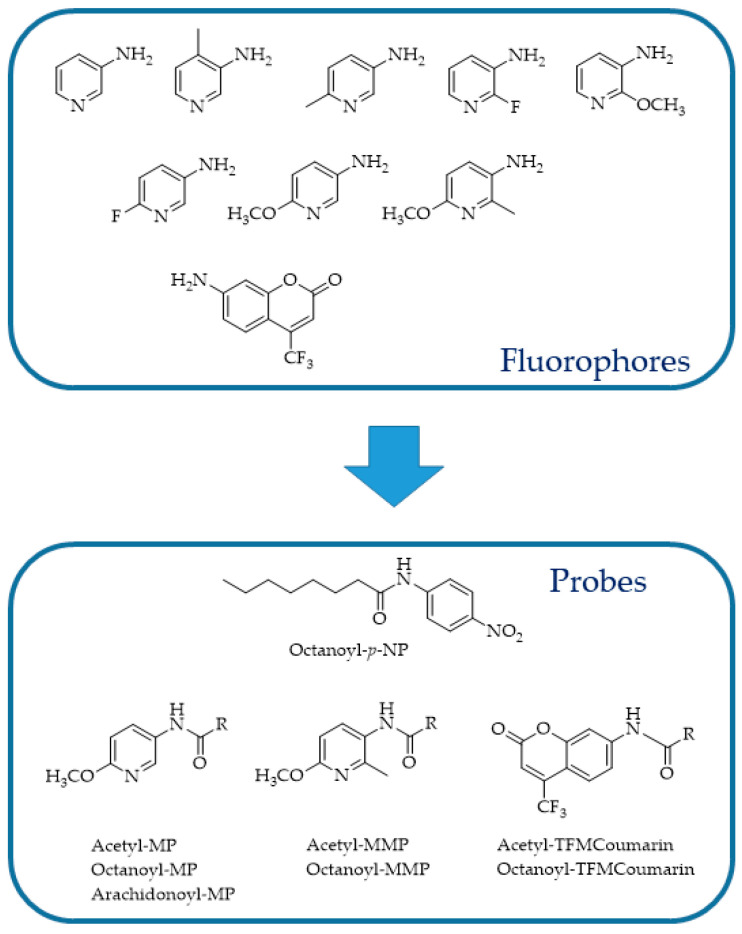
Structures of fluorescent reporters and substrates [120].

In 2006, Wang et al. [118] adapted the assay developed by Ramarao et al. [100] to an HTS format and screened a large library of small organic compounds, identifying a number of novel FAAH inhibitors. To obtain an HTS method, the author assessed different reaction conditions such as substrate concentrations, reaction times, and enzyme amounts. Microsomes expressing FAAH, prepared from Chinese hamster ovary DukX/A2 cells instead of the purified enzyme, were used. The obtained data demonstrated that this fluorescent assay was sufficiently robust, efficient, and low cost for the screening and identification of FAAH inhibitory molecules. Similarly, Kage et al. [119] designed a new fluorogenic FAAH probe by substituting the AA with decanoic acid and validated it in the same experimental conditions described for AAMCA (Figure 14). The reason that prompted the authors to make this change is that AAMCA suffers from low solubility and stability due to the longer hydrocarbon chain and potentially labile *cis* olefins. Huang et al. [120] described the development of a class of pyridine derivatives as fluorescent probes for FAAH, with 3- or 5-aminopyridines substituted (Figure 15), and devised a novel, simple, and highly sensitive fluorescent assay for FAAH activity.

This assay relies on detecting the fluorescence emitted by a substituted 3- or 5-aminopyridine after amide hydrolysis. According to the authors, substituted aminopyridines possess many advantages such as a higher fluorescence, better aqueous solubility, and smaller size. Furthermore, the nitrogen situated on the pyridine ring might act as an electron donor or be potentially engaged in hydrogen bonding, thereby aiding in the hydrolysis of substrates by amide hydrolases. The novel fluorescent assays set up using as substrate aminopyridines were at least 50 times better than those previously published in the literature, either fluorescent or colorimetric assays. Moreover, the assessment of the proposed probes on human liver microsomes allowed for the discovery of at least one new amide hydrolase for long-chain fatty acid amides expressed by the microsomes, stimulating further research in this field.

**Figure 16:**
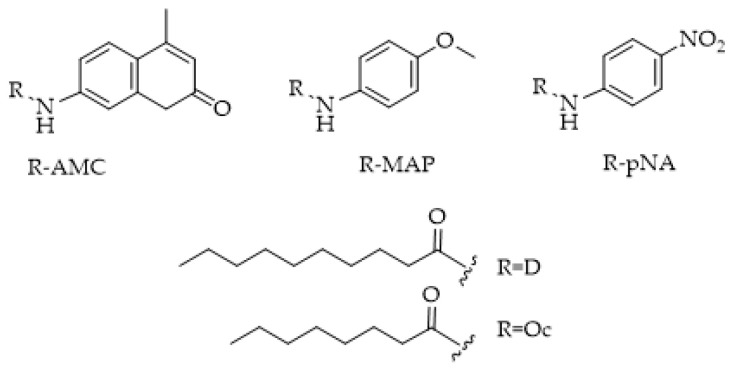
Structures of p-nitroaniline (R-pNA), 5-amino-2-methoxypyridine (R-MAP), and 7-amino-4-methylcoumarin (R-AMC) containing substrates of FAAH. D = decanoyl; Oc = octanoyl.

FAAH substrates initially contained AA, producing potent ligands such as *N*-(4-nitrophenyl)arachidonylamide (AA-pNA) [121], *N*-(4-methylcoumarin)arachidonylamide (AA-AMC) [100,118], and *N*-(6-methoxypyridin-3-yl)arachidonylamide (AA-MAP) [120]. These ligands, however, showed problems related to their relatively low water solubility and reduced stability due to the extended hydrocarbon chain (C20) and potentially labile cis double bonds, respectively [119,120]. Dato et al. [122] proposed *N*-decanoyl-substituted 5-amino-2-methoxypyridine (D-MAP, Figure 16) as a new amide substrate to overcome the long and polyunsaturated acyl chain-stability problems. The choice of decanoic acid chain was based on the demonstrated ability of FAAH to also hydrolyze medium chain amides, with the best performance using decanoic derivatives [121]. Moreover, the 5-amino-2-methoxypyridine (MAP) fluorophore was selected instead of 7-amino-4-methylcoumarin (AMC) due to its superior sensitivity and water solubility [120]. The authors also characterized their probe in comparison to the literature-known substrates *N*-(6-methoxypyridin-3-yl)octanamide (Oc-MAP) [120], *N*-(4-nitrophenyl)decanamide (D-pNA) [115], and *N*-(4-nitrophenyl)octanamide (Oc-pNA) [115] with respect to the aqueous solubility and substrate properties. As expected, D-MAP demonstrated major affinity toward FAAH (eight-fold higher specificity constant than Oc-MAP) and increased aqueous solubility if compared to the respective p-nitroaniline derivatives (D-pNA and Oc-pNA). Moreover, the high sensitivity of the D-MAP assay allows for the use of very low enzyme amounts (1 μg mL^−1^) if compared with previous methods.

**Figure 17:**
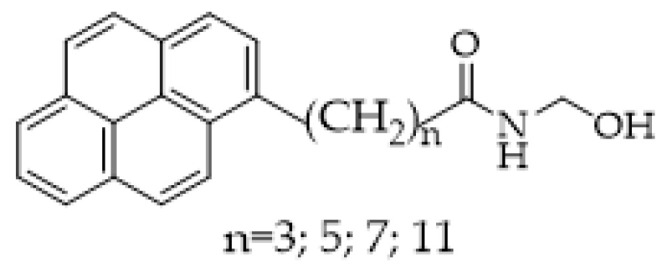
Structures of putative pyrenyl-containing substrates of FAAH. n = 3 *N*-(2-hydroxyethyl)-4-pyren-1-ylbutanamide; n = 5 *N*-(2-hydroxyethyl)-4-pyren-1-ylhexanamide; n = 7 *N*-(2-hydroxyethyl)-4-pyren-1-yloctanamide; n = 11 *N*-(2-hydroxyethyl)-4-pyren-1-yldodecanamide.

Foster and al. [123] proposed a novel approach to the design of FAAH fluorogenic probes, maintaining the integrity of the amide part of the enzyme natural substrate and introducing the fluorophore in place of the acyl chain. With this intent, four new ethanolamides of pyrenylalkanoic acids, characterized by a different distance between the pyrenyl and amide groups, have been synthesized and evaluated as FAAH probes (Figure 17). HPLC coupled with a fluorescence detector to evaluate pyrene release was then used to determine FAAH, avoiding further sample cleanup. Triton X-100 was added to the assay buffer due to the low water solubility of the probes. From the proposed compounds, butan- and exan-amides showed the best performances, while octanoic and decanoic derivatives were not hydrolyzed by FAAH.

**Figure 18:**
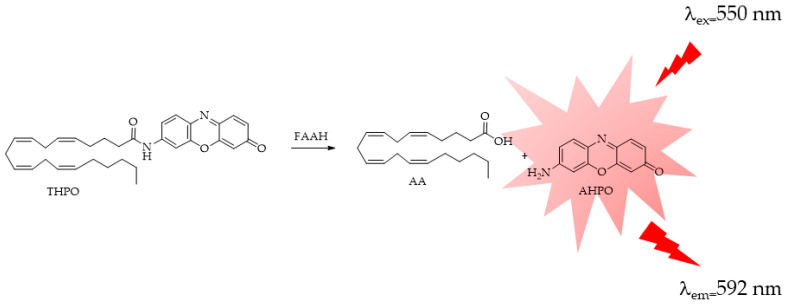
FAAH-responsive fluorescent probe THPO: (THPO = arachidonic acid derivative of 7-amino-3H-phenoxazin-3-one (AHPO).

In a recent study, Tian X. et al. [124] developed a new probe, named THPO, with the aim of monitoring endogenous FAAH. In this case, the AA, even if characterized by the stability and water-solubility problems described before, was introduced as a specific recognition moiety for FAAH (Figure 18). 7-amino-3H-phenoxazin-3-one (AHPO), a long-wavelength fluorescent probe, was then chosen as the fluorescent dye due the need of a red-shifted emitting fluorophore aiming to reduce noise and interfering signals linked to cells experiments. THPO offers several advantages: firstly, its metabolite AHPO not only emits light in the red spectrum (λ_ex_/λ_em_ = 550/592 nm) but also boasts a high fluorescence quantum yield. Secondly, THPO demonstrates remarkable specificity and sensitivity towards FAAH among a range of hydrolases. Thirdly, THPO can be utilized for monitoring and imaging FAAH in living cells. In summary, THPO has the potential to be a valuable tool for swiftly assessing the FAAH activity in complex systems and for establishing a visual HTS method for FAAH inhibitors.

**Figure 19:**
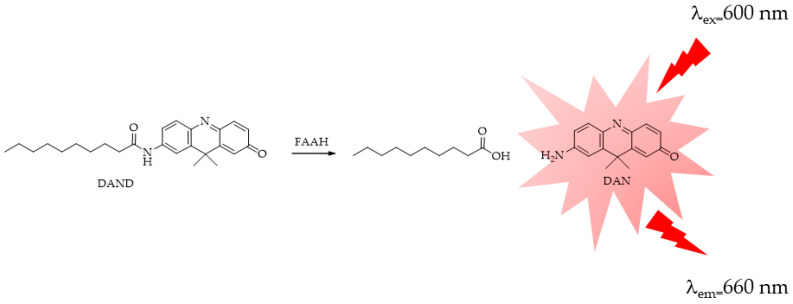
FAAH-responsive fluorescent probe DAND: DAND = decanoic acid derivative of 7-amino-9,9-dimethylacridin-2(9H)-one (DAN).

More recently, Tian M. et al. [86], aiming at developing a visual HTS method that would allow for the real-time detection and imaging of the FAAH activity in living cells or complex bio-systems, designed a highly selective and sensitive FAAH-activated near-infrared (NIR) fluorescent probe. According to the authors of the study, NIR fluorescent probes are preferable owing to the reduced background absorbance of the activation wavelength and the significantly reduced interference and absorption of the emission wavelength. Enzyme-activated fluorescent probe DAND (Figure 19) was designed and synthesized based on the catalytic characteristics of FAAH by introducing decanoic acid as the specific recognition moiety in the fluorophore of 7-amino-9,9-dimethylacridin-2(9H)-one (DAN). DAND was also successfully used for the real-time imaging of FAAH in living cells.

**Figure 20:**
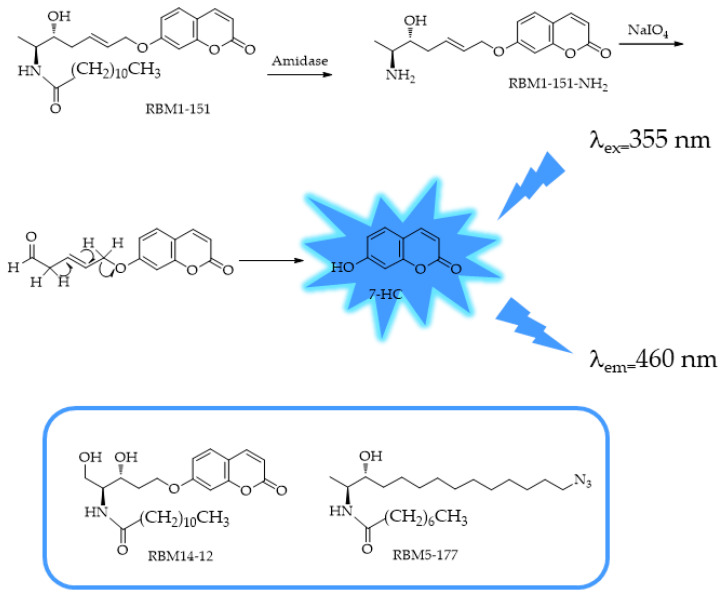
Scheme of production of 7-hydroxyl coumarin (7-HC) from RBM1-151 by amide hydrolysis and further in situ oxidation and β-elimination. In the box are the original compounds that inspired RBM1-151: RBM5-177 and RBM14-C12.

In 2024, Casasampere et al. [125] reported the functional characterization of coumarin 1-deoxydihydroceramide, RBM1-151, as a new substrate of amidases, including FAAH and NAAA (Figure 20).

The original compounds that inspired RBM1-151, RBM5-177 and RBM14-C12, were developed as novel fluorogenic sensors for the determination of sphingosine-1-phosphate liase activity in cell lysates [126]. The method is based on the evaluation of the fluorescent 7-hydroxyl coumarin (7-HC) released from the aldehyde, resulting from amidase cleavage of the substrate, by in situ oxidation and β-elimination. RBM1-151 is not hydrolyzed by any other ceramidase, but it is deamidated by FAAH and NAAA. By combining the fluorogenic amidase substrate RBM1-151, the authors have developed a system to monitor AC, FAAH, and NAAA not only in intact cells but also for HTS methods for the three enzymes.

### 4.4. NAAA

**Figure 21:**
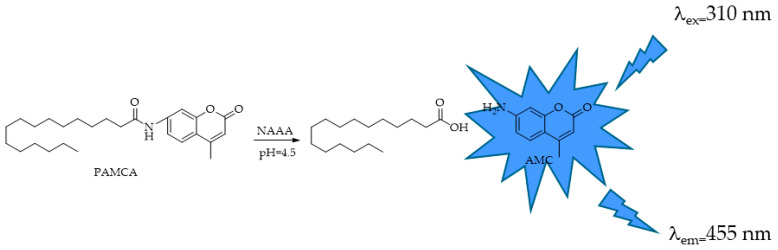
NAAA-responsive fluorescent probe *N*-(4-methyl coumarin) palmitamide (PAMCA).

In addition to FAAH, another NAE-hydrolyzing amidohydrolase active only at an acidic pH was identified, NAAA, characterized by an optimal pH around 5 and the preference of PEA, an anti-inflammatory mediator, to other NAEs. Among the hydrolytic enzymes of the ECS, NAAA is the least studied to date, and there are only few suitable HTS assay methods for determining its activity. The assay procedures used to screen and characterize NAAA activity employ either radioactive products [71,74,127] or mass spectrometry [128]. These testing methods are expensive and poorly suited to HTS. The fluorometric methods used for NAAA are the same as those used for FAAH but adapted to the conditions for the enzyme. In 2012, West et al. [129] developed the fluorogenic PEA analog of AAMCA, *N*-(4-methyl coumarin) palmitamide (PAMCA, Figure 21), which is hydrolyzed to fluorescent 7-amino-4-methyl coumarin (AMC) and palmitic acid by NAAA at pH 4.5. The assay procedure is analogous to that used in the fluorescence-based assays for FAAH and MAGL and is performed on the protein extract of HEK293 cells expressing human recombinant NAAA (*h*NAAA). The same method has been applied by Vago and coworkers on the isolated *h*NAAA [29].

Yang et al. [130] reported a fluorometric measurement of NAAA activity, similar to that previously reported with PAMCA as the fluorogenic probe [129,131] with slight modifications, used to evaluate the inhibitory potency of natural products. In 2024, Casasampere et al. [125] reported the use of a coumarin derivative, RBM1-151, as a novel substrate of amidases, including NAAA, as mentioned above.

## 5. Application of Fluorescent Probes in Living Cells

To date, despite an in-depth analysis of the current literature, only few methods have been proposed for the evaluation of the activity of the ECS hydrolytic enzymes in living cells. Starting from the aforementioned probes, THAPO (Figure 18), thanks to its high selectivity together with a good cell permeability and bio-compatibility, displayed the ability to detect FAAH activity in neural SHSY-5Y living cells, showing a consistent reduction of the fluorescence signal in the presence of a selective inhibitor (URB597) [124]. In addition, the NIR fluorescent probe DAND (Figure 19) has been also proposed for the detection of FAAH activity in living cells. The authors firstly explored the selectivity of DAND, comparing the fluorescence signal in the presence of FAAH with that produced by other metabolic enzymes comprising lipases and hydrolases. In a second step, the effects of selective inhibitors, URB597 for FAAH and other inhibitors specific for the hydrolases and lipases assessed, on the fluorescence signal were evaluated, revealing a decrease in fluorescence only in the presence of URB597. Lastly, DAND signal stability in the presence of common endogenous substances such as amino acids, myristic acid, and glucose or ions was evaluated. Once established, the high selectivity and good stability of the probe in complex bio-matrices, with the fluorescence signal produced in BV2, C6, and U251 living glial cells, was evaluated in the presence or absence of URB597, demonstrating a decrease in fluorescence production in the presence of the selective inhibitor [132]. Even the probe RBM1-151 (Figure 20), proposed by Casasampere and coworkers for the detection of lipid amidases, allowed for the determination of the activity of acid ceramidase, FAAH, and NAAA in intact cells if used in combination with selective inhibitors. The proposed fluorescence method has the advantage to highlight the modulation of the activities of these three enzymes in response to large-scale experiments, in HTS format (96-well plate) with a single substrate. Unfortunately, the requirement of an additional oxidative step, performed with sodium periodate, after umbelliferon release to obtain the fluorescence signal limits its use in living cells [125].

**Figure 22:**
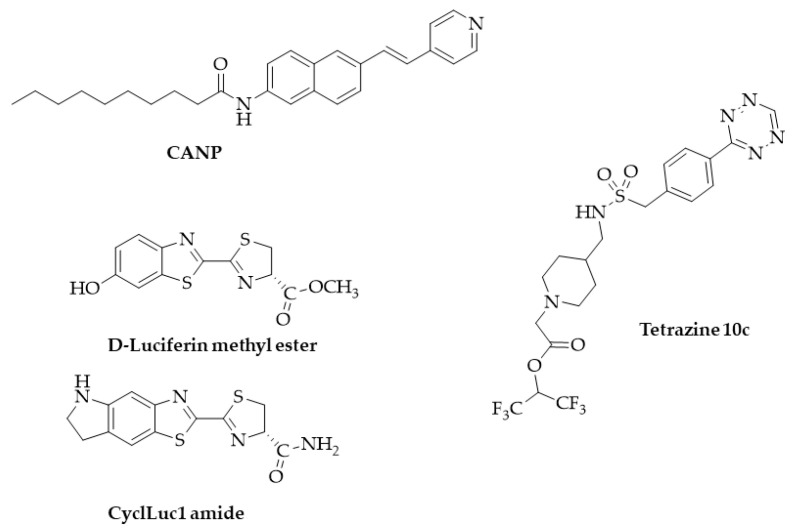
Structure of probes used for enzyme activity evaluation in living cells.

In addition, a novel NIR two-photon ratiometric, fluorescent probe named CANP (Figure 22), based on a naphthylvynylpyridine monofluorophore released from CANP by the enzymatic cleavage of the amide bound, has been recently proposed for FAAH activity imaging in live neurons. The probe was completely characterized and validated on the recombinant enzyme in the presence or absence of the inhibitor URB597. Furthermore, its cytotoxicity and biocompatibility were assessed on neurons. A first experiment of co-localization confirmed the accumulation of the CANP signal in the cytosol where FAAH is active, then a probe was used to image the dynamics of endogenous FAAH levels and activity in response to different stimuli or in the presence of URB597 [133].

A new fluorescent probe to evaluate MAGL activity in living cells has been also recently proposed. From a small library of monosubstituted and unsymmetrical disubstituted tetrazines, the tetrazine 10c (Figure 22) displayed a favorable combination of kinetics, small size, and hydrophilicity and was selected for experiments in living cells. This new probe, in combination with the TCO-TAMRA labeling system, allowed for the labeling of active MAGL in human brain pericytes [134].

Very recently, Mohammad et al. presented luciferin derivatives, such as D-luciferin methyl ester and CycLuc1 amide (Figure 22), to “illuminate the activity of multiple serine hydrolases” in living cells [135]. The authors validated the proposed probes for MAGL activity detection on prostate cancer (PC3) and glioma (U87) cells transfected with firefly luciferase. For FAAH validation, instead, HEK293 cells overexpressing the enzyme and expressing luciferase were used. The inability of the probes to evaluate the ABHD6 activity was also demonstrated. However, the poor selectivity of the proposed probes limits its application in living cells expressing comparable amounts of the different target enzymes.

Despite reports in the literature of an increasing number of probes used for the development of HTS methods assessing ECS hydrolytic enzyme activities in the presence or absence of inhibitors, only a few of them enable the evaluation of these enzyme activities in living cells. Moreover, most of the proposed methods cannot be used for time-course or continuous analysis due to the multiple steps required for fluorescence detection, such as oxidation, protein extraction, or electron microscopy application.

## 6. Advantages and Disadvantages

Enzyme-activated fluorescent probes are likely the most extensively utilized detection method in enzymatic drug screening and disease diagnosis, primarily because they are suitable for HTS and provide great performance and flexibility. The high sensitivities of these methods, which are 100 to 1000 times greater than that of absorption photometry, are especially useful in studies where only small amounts of samples are available and permits the incubation of enzymes in dilute solutions under conditions where the kinetics and the influence of inhibitors or activators are often studied with more validity. The incubation time is short, and the monitoring of the product over time is continuous; the measurement is directly in the reaction medium, and a high number of samples and replicates are processed simultaneously (96 wells/plate). Finally, disposable materials such as pipette tips and microplates are readily available, allowing plate set-up and measurement to be completed in less than one hour. Furthermore, the automatic calculation of the activity rates in relative units of fluorescence/min facilitates the interpretation of the results. Due to the absence of a detection reagent requirement, fluorescence-based assays exhibit high stability, enabling batch processing of plates as well as the capability for plates to be read multiple times.

In contrast, although fluorescence-based assays offer powerful tools for biochemical and biomedical research, one must be aware of their limitations. As described before, auto-bio-fluorescence, photobleaching, or environmental factors can interfere with the analyses. Moreover, different fluorophores emit distinct wavelengths of light, enabling researchers to employ fluorescence assays for multiplexing and the simultaneous measurement of multiple targets on a single plate.

Addressing these challenges through improved experimental protocols, advanced instrumentation, and alternative assay designs will further enhance the utility and reliability of fluorescence-based techniques in scientific investigations.

Future studies on endocannabinoid hydrolytic enzymes should prioritize elucidating their substrate specificity across different physiological contexts. Investigating the kinetics of enzyme-catalyzed hydrolysis and their modulation by endogenous and exogenous factors will enhance our understanding of their regulatory mechanisms. Furthermore, exploring the structural basis of enzyme–substrate interactions and identifying novel inhibitors or activators could uncover therapeutic strategies for manipulating the endocannabinoid system in various health conditions.

## Data Availability

Data sharing not applicable.

## References

[1] Maccarrone M., Finazzi-Agró A. (2003). The endocannabinoid system, anandamide and the regulation of mammalian cell apoptosis. Cell Death Differ..

[2] Mock E.D., Gagestein B., van der Stelt M. (2023). Anandamide and other N-acylethanolamines: A class of signaling lipids with therapeutic opportunities. Prog. Lipid Res..

[3] Teplick J.G., Wallner R.J., Levine A.H., Haskin M.E., Teplick S.K. (1979). Isolated dextrogastria: Report of two cases. AJR Am. J. Roentgenol..

[4] Russell L., Condo K., DeFlorville T. (2024). Nutrition, endocannabinoids, and the use of cannabis: An overview for the nutrition clinician. Nutr. Clin. Pract..

[5] Ottria R., Della Porta M., Xynomilakis O., Casati S., Cazzola R., Ciuffreda P. (2024). Lipids and lipid signaling molecules in human milk and infant formula, a chemical characterization of relevant biochemical components. J. Nutr. Biochem..

[6] Ellermann M. (2023). Emerging mechanisms by which endocannabinoids and their derivatives modulate bacterial populations within the gut microbiome. Adv. Drug Alcohol Res..

[7] Kurlyandchik I., Lauche R., Tiralongo E., Warne L.N., Schloss J. (2022). Plasma and interstitial levels of endocannabinoids and N-acylethanolamines in patients with chronic widespread pain and fibromyalgia: A systematic review and meta-analysis. Pain Rep..

[8] Wu Y., Han C., Luo R., Cai W., Xia Q., Jiang R., Ferdek P.E., Liu T., Huang W. (2023). Molecular mechanisms of pain in acute pancreatitis: Recent basic research advances and therapeutic implications. Front. Mol. Neurosci..

[9] Pezzilli R., Ciuffreda P., Ottria R., Ravelli A., Melzi d’Eril G., Barassi A. (2017). Serum endocannabinoids in assessing pain in patients with chronic pancreatitis and in those with pancreatic ductal adenocarcinoma. Scand. J. Gastroenterol..

[10] Clouse G., Penman S., Hadjiargyrou M., Komatsu D.E., Thanos P.K. (2022). Examining the role of cannabinoids on osteoporosis: A review. Arch. Osteoporos..

[11] Casati S., Giannasi C., Minoli M., Niada S., Ravelli A., Angeli I., Mergenthaler V., Ottria R., Ciuffreda P., Orioli M. (2020). Quantitative Lipidomic Analysis of Osteosarcoma Cell-Derived Products by UHPLC-MS/MS. Biomolecules.

[12] Carnovali M., Ottria R., Pasqualetti S., Banfi G., Ciuffreda P., Mariotti M. (2016). Effects of bioactive fatty acid amide derivatives in zebrafish scale model of bone metabolism and disease. Pharmacol. Res..

[13] Ottria R., Cappelletti L., Ravelli A., Mariotti M., Gigli F., Romagnoli S., Ciuffreda P., Banfi G., Drago L. (2016). Plasma endocannabinoid behaviour in total knee and hip arthroplasty. J. Biol. Regul. Homeost. Agents.

[14] Di Marzo V., Fontana A., Cadas H., Schinelli S., Cimino G., Schwartz J.C., Piomelli D. (1994). Formation and inactivation of endogenous cannabinoid anandamide in central neurons. Nature.

[15] Piomelli D., Beltramo M., Glasnapp S., Lin S.Y., Goutopoulos A., Xie X.Q., Makriyannis A. (1999). Structural determinants for recognition and translocation by the anandamide transporter. Proc. Natl. Acad. Sci. USA.

[16] Cravatt B.F., Giang D.K., Mayfield S.P., Boger D.L., Lerner R.A., Gilula N.B. (1996). Molecular characterization of an enzyme that degrades neuromodulatory fatty-acid amides. Nature.

[17] Dinh T.P., Carpenter D., Leslie F.M., Freund T.F., Katona I., Sensi S.L., Kathuria S., Piomelli D. (2002). Brain monoglyceride lipase participating in endocannabinoid inactivation. Proc. Natl. Acad. Sci. USA.

[18] Ottria R., Ravelli A., Gigli F., Ciuffreda P. (2014). Simultaneous ultra-high performance liquid chromathograpy-electrospray ionization-quadrupole-time of flight mass spectrometry quantification of endogenous anandamide and related N-acylethanolamides in bio-matrices. J. Chromatogr. B Analyt. Technol. Biomed. Life Sci..

[19] Marchioni C., de Souza I.D., Acquaro V.R., de Souza Crippa J.A., Tumas V., Queiroz M.E.C. (2018). Recent advances in LC-MS/MS methods to determine endocannabinoids in biological samples: Application in neurodegenerative diseases. Anal. Chim. Acta.

[20] Vago R., Ravelli A., Bettiga A., Casati S., Lavorgna G., Benigni F., Salonia A., Montorsi F., Orioli M., Ciuffreda P. (2020). Urine Endocannabinoids as Novel Non-Invasive Biomarkers for Bladder Cancer at Early Stage. Cancers.

[21] Zoerner A.A., Gutzki F.-M., Batkai S., May M., Rakers C., Engeli S., Jordan J., Tsikas D. (2011). Quantification of endocannabinoids in biological systems by chromatography and mass spectrometry: A comprehensive review from an analytical and biological perspective. Biochim. Biophys. Acta.

[22] Ottria R., Casati S., Rota P., Ciuffreda P. (2022). 2-Arachidonoylglycerol Synthesis: Facile and Handy Enzymatic Method That Allows to Avoid Isomerization. Molecules.

[23] Ottria R., Casati S., Ciuffreda P. (2012). Optimized synthesis and characterization of N-acylethanolamines and O-acylethanolamines, important family of lipid-signalling molecules. Chem. Phys. Lipids.

[24] Piomelli D., Giuffrida A., Calignano A., Rodríguez de Fonseca F. (2000). The endocannabinoid system as a target for therapeutic drugs. Trends Pharmacol. Sci..

[25] Ramer R., Wittig F., Hinz B. (2021). The Endocannabinoid System as a Pharmacological Target for New Cancer Therapies. Cancers.

[26] Jaiswal S., Ayyannan S.R. (2021). Anticancer Potential of Small-Molecule Inhibitors of Fatty Acid Amide Hydrolase and Monoacylglycerol Lipase. Chem. Med. Chem..

[27] van Egmond N., Straub V.M., van der Stelt M. (2021). Targeting Endocannabinoid Signaling: FAAH and MAG Lipase Inhibitors. Annu. Rev. Pharmacol. Toxicol..

[28] Lauria S., Perrotta C., Casati S., Di Renzo I., Ottria R., Eberini I., Palazzolo L., Parravicini C., Ciuffreda P. (2018). Design, synthesis, molecular modelling and in vitro cytotoxicity analysis of novel carbamate derivatives as inhibitors of Monoacylglycerol lipase. Bioorg. Med. Chem..

[29] Vago R., Bettiga A., Salonia A., Ciuffreda P., Ottria R. (2017). Development of new inhibitors for N-acylethanolamine-hydrolyzing acid amidase as promising tool against bladder cancer. Bioorg. Med. Chem..

[30] Lazarević J., Šmelcerović A., Zvezdanović J., Yancheva D., Casati S., Ottria R., Ciuffreda P. (2020). Lipid peroxidation inhibition study: A promising case of 1,3-di(1,1′-biphenyl-3-yl)urea. Chem. Biol. Interact..

[31] Jain S., Bisht A., Verma K., Negi S., Paliwal S., Sharma S. (2022). The role of fatty acid amide hydrolase enzyme inhibitors in Alzheimer’s disease. Cell Biochem. Funct..

[32] Kashyap A., Kumar S., Dutt R. (2022). A Review on Structurally Diversified Synthesized Molecules as Monoacylglycerol Lipase Inhibitors and their Therapeutic uses. Curr. Drug Res. Rev..

[33] Bononi G., Poli G., Rizzolio F., Tuccinardi T., Macchia M., Minutolo F., Granchi C. (2021). An updated patent review of monoacylglycerol lipase (MAGL) inhibitors (2018-present). Expert Opin. Ther. Pat..

[34] Kicman A., Pędzińska-Betiuk A., Kozłowska H. (2021). The potential of cannabinoids and inhibitors of endocannabinoid degradation in respiratory diseases. Eur. J. Pharmacol..

[35] Mikkelsen J.D., Aripaka S.S., Egilmez C.B., Pazarlar B.A. (2024). Binding of the monoacylglycerol lipase (MAGL) radiotracer 3HT-401 in the rat brain after status epilepticus. Neurochem. Int..

[36] Hou L., Rong J., Haider A., Ogasawara D., Varlow C., Schafroth M.A., Mu L., Gan J., Xu H., Fowler C.J. (2021). Positron Emission Tomography Imaging of the Endocannabinoid System: Opportunities and Challenges in Radiotracer Development. J. Med. Chem..

[37] Cécyre B., Monette M., Beudjekian L., Casanova C., Bouchard J.-F. (2014). Localization of diacylglycerol lipase alpha and monoacylglycerol lipase during postnatal development of the rat retina. Front. Neuroanat..

[38] Rivera P., Arrabal S., Cifuentes M., Grondona J.M., Pérez-Martín M., Rubio L., Vargas A., Serrano A., Pavón F.J., Suárez J. (2014). Localization of the cannabinoid CB1 receptor and the 2-AG synthesizing (DAGLα) and degrading (MAGL, FAAH) enzymes in cells expressing the Ca(2+)-binding proteins calbindin, calretinin, and parvalbumin in the adult rat hippocampus. Front. Neuroanat..

[39] Zhou L., Tian M., Zhang B., Cao X., Huo X., Yang F., Cao P., Feng L., Ma X., Tian X. (2024). Lysosome targeting fluorescent probe for NAAA imaging and its applications in the drug development for anti-inflammatory. Int. J. Biol. Macromol..

[40] Yapa U., Prusakiewicz J.J., Wrightstone A.D., Christine L.J., Palandra J., Groeber E., Wittwer A.J. (2012). High-performance liquid chromatography-tandem mass spectrometry assay of fatty acid amide hydrolase (FAAH) in blood: FAAH inhibition as clinical biomarker. Anal. Biochem..

[41] Angelucci C.B., Giacominelli-Stuffler R., Maccarrone M. (2023). Fluorimetric Assay of FAAH Activity. Methods Mol. Biol..

[42] Jung K.-M., Piomelli D. (2023). Assay of Monoacylglycerol Lipase Activity. Methods Mol. Biol..

[43] Blankman J.L., Simon G.M., Cravatt B.F. (2007). A comprehensive profile of brain enzymes that hydrolyze the endocannabinoid 2-arachidonoylglycerol. Chem. Biol..

[44] Bertrand T., Augé F., Houtmann J., Rak A., Vallée F., Mikol V., Berne P.F., Michot N., Cheuret D., Hoornaert C. (2010). Structural basis for human monoglyceride lipase inhibition. J. Mol. Biol..

[45] Labar G., Bauvois C., Muccioli G.G., Wouters J., Lambert D.M. (2007). Disulfiram is an inhibitor of human purified monoacylglycerol lipase, the enzyme regulating 2-arachidonoylglycerol signaling. ChemBioChem.

[46] Long J.Z., Cravatt B.F. (2011). The metabolic serine hydrolases and their functions in mammalian physiology and disease. Chem. Rev..

[47] Karlsson M., Contreras J.A., Hellman U., Tornqvist H., Holm C. (1997). cDNA cloning, tissue distribution, and identification of the catalytic triad of monoglyceride lipase. Evolutionary relationship to esterases, lysophospholipases, and haloperoxidases. J. Biol. Chem..

[48] Sugiura T., Kondo S., Sukagawa A., Nakane S., Shinoda A., Itoh K., Yamashita A., Waku K. (1995). 2-Arachidonoylglycerol: A possible endogenous cannabinoid receptor ligand in brain. Biochem. Biophys. Res. Commun..

[49] Dinh T.P., Kathuria S., Piomelli D. (2004). RNA interference suggests a primary role for monoacylglycerol lipase in the degradation of the endocannabinoid 2-arachidonoylglycerol. Mol. Pharmacol..

[50] Deng H., Li W. (2020). Therapeutic potential of targeting α/β-Hydrolase domain-containing 6 (ABHD6). Eur. J. Med. Chem..

[51] Vandevoorde S., Saha B., Mahadevan A., Razdan R.K., Pertwee R.G., Martin B.R., Fowler C.J. (2005). Influence of the degree of unsaturation of the acyl side chain upon the interaction of analogues of 1-arachidonoylglycerol with monoacylglycerol lipase and fatty acid amide hydrolase. Biochem. Biophys. Res. Commun..

[52] Alhouayek M., Masquelier J., Muccioli G.G. (2014). Controlling 2-arachidonoylglycerol metabolism as an anti-inflammatory strategy. Drug Discov. Today.

[53] Nomura D.K., Long J.Z., Niessen S., Hoover H.S., Ng S.-W., Cravatt B.F. (2010). Monoacylglycerol lipase regulates a fatty acid network that promotes cancer pathogenesis. Cell.

[54] Muccioli G.G., Xu C., Odah E., Cudaback E., Cisneros J.A., Lambert D.M., López Rodríguez M.L., Bajjalieh S., Stella N. (2007). Identification of a novel endocannabinoid-hydrolyzing enzyme expressed by microglial cells. J. Neurosci..

[55] Kind L., Kursula P. (2019). Structural properties and role of the endocannabinoid lipases ABHD6 and ABHD12 in lipid signalling and disease. Amino Acids.

[56] Baggelaar M.P., van Esbroeck A.C.M., van Rooden E.J., Florea B.I., Overkleeft H.S., Marsicano G., Chaouloff F., van der Stelt M. (2017). Chemical Proteomics Maps Brain Region Specific Activity of Endocannabinoid Hydrolases. ACS Chem. Biol..

[57] Thomas G., Betters J.L., Lord C.C., Brown A.L., Marshall S., Ferguson D., Sawyer J., Davis M.A., Melchior J.T., Blume L.C. (2013). The Serine Hydrolase ABHD6 Is a Critical Regulator of the Metabolic Syndrome. Cell Rep..

[58] Navia-Paldanius D., Savinainen J.R., Laitinen J.T. (2012). Biochemical and pharmacological characterization of human α/β-hydrolase domain containing 6 (ABHD6) and 12 (ABHD12). J. Lipid Res..

[59] Cao J.K., Kaplan J., Stella N. (2019). ABHD6: Its Place in Endocannabinoid Signaling and Beyond. Trends Pharmacol. Sci..

[60] Alhouayek M., Masquelier J., Cani P.D., Lambert D.M., Muccioli G.G. (2013). Implication of the anti-inflammatory bioactive lipid prostaglandin D2-glycerol ester in the control of macrophage activation and inflammation by ABHD6. Proc. Natl. Acad. Sci. USA.

[61] Zhao S., Poursharifi P., Mugabo Y., Levens E.J., Vivot K., Attane C., Iglesias J., Peyot M.-L., Joly E., Madiraju S.R.M. (2015). α/β-Hydrolase domain-6 and saturated long chain monoacylglycerol regulate insulin secretion promoted by both fuel and non-fuel stimuli. Mol. Metab..

[62] Zhao S., Mugabo Y., Ballentine G., Attane C., Iglesias J., Poursharifi P., Zhang D., Nguyen T.A., Erb H., Prentki R. (2016). α/β-Hydrolase Domain 6 Deletion Induces Adipose Browning and Prevents Obesity and Type 2 Diabetes. Cell Rep..

[63] Max D., Hesse M., Volkmer I., Staege M.S. (2009). High expression of the evolutionarily conserved alpha/beta hydrolase domain containing 6 (ABHD6) in Ewing tumors. Cancer Sci..

[64] Joshi A., Shaikh M., Singh S., Rajendran A., Mhetre A., Kamat S.S. (2018). Biochemical characterization of the PHARC-associated serine hydrolase ABHD12 reveals its preference for very-long-chain lipids. J. Biol. Chem..

[65] Saghatelian A., McKinney M.K., Bandell M., Patapoutian A., Cravatt B.F. (2006). A FAAH-regulated class of N-acyl taurines that activates TRP ion channels. Biochemistry.

[66] Chebrou H., Bigey F., Arnaud A., Galzy P. (1996). Study of the amidase signature group. Biochim. Biophys. Acta.

[67] Lichtman A.H., Hawkins E.G., Griffin G., Cravatt B.F. (2002). Pharmacological activity of fatty acid amides is regulated, but not mediated, by fatty acid amide hydrolase in vivo. J. Pharmacol. Exp. Ther..

[68] Kathuria S., Gaetani S., Fegley D., Valiño F., Duranti A., Tontini A., Mor M., Tarzia G., La Rana G., Calignano A. (2003). Modulation of anxiety through blockade of anandamide hydrolysis. Nat. Med..

[69] Cravatt B.F., Saghatelian A., Hawkins E.G., Clement A.B., Bracey M.H., Lichtman A.H. (2004). Functional disassociation of the central and peripheral fatty acid amide signaling systems. Proc. Natl. Acad. Sci. USA.

[70] Boger D.L., Sato H., Lerner A.E., Hedrick M.P., Fecik R.A., Miyauchi H., Wilkie G.D., Austin B.J., Patricelli M.P., Cravatt B.F. (2000). Exceptionally potent inhibitors of fatty acid amide hydrolase: The enzyme responsible for degradation of endogenous oleamide and anandamide. Proc. Natl. Acad. Sci. USA.

[71] Tsuboi K., Sun Y.-X., Okamoto Y., Araki N., Tonai T., Ueda N. (2005). Molecular characterization of N-acylethanolamine-hydrolyzing acid amidase, a novel member of the choloylglycine hydrolase family with structural and functional similarity to acid ceramidase. J. Biol. Chem..

[72] Rossocha M., Schultz-Heienbrok R., von Moeller H., Coleman J.P., Saenger W. (2005). Conjugated bile acid hydrolase is a tetrameric N-terminal thiol hydrolase with specific recognition of its cholyl but not of its tauryl product. Biochemistry.

[73] Zhao L.-Y., Tsuboi K., Okamoto Y., Nagahata S., Ueda N. (2007). Proteolytic activation and glycosylation of N-acylethanolamine-hydrolyzing acid amidase, a lysosomal enzyme involved in the endocannabinoid metabolism. Biochim. Biophys. Acta.

[74] Saturnino C., Petrosino S., Ligresti A., Palladino C., de Martino G., Bisogno T., Di Marzo V. (2010). Synthesis and biological evaluation of new potential inhibitors of N-acylethanolamine hydrolyzing acid amidase. Bioorg. Med. Chem. Lett..

[75] Solorzano C., Antonietti F., Duranti A., Tontini A., Rivara S., Lodola A., Vacondio F., Tarzia G., Piomelli D., Mor M. (2010). Synthesis and structure-activity relationships of N-(2-oxo-3-oxetanyl)amides as N-acylethanolamine-hydrolyzing acid amidase inhibitors. J. Med. Chem..

[76] Liu H.-W., Chen L., Xu C., Li Z., Zhang H., Zhang X.-B., Tan W. (2018). Recent progresses in small-molecule enzymatic fluorescent probes for cancer imaging. Chem. Soc. Rev..

[77] Wang R., Chen J., Gao J., Chen J.-A., Xu G., Zhu T., Gu X., Guo Z., Zhu W.-H., Zhao C. (2019). A molecular design strategy toward enzyme-activated probes with near-infrared I and II fluorescence for targeted cancer imaging. Chem. Sci..

[78] Fernández-Ruiz R. (2022). TXRF spectrometry in the bioanalytical sciences: A brief review. X-ray Spectrom..

[79] Hua L., Wang D., Wang K., Wang Y., Gu J., Zhang Q., You Q., Wang L. (2023). Design of Tracers in Fluorescence Polarization Assay for Extensive Application in Small Molecule Drug Discovery. J. Med. Chem..

[80] Fang B., Shen Y., Peng B., Bai H., Wang L., Zhang J., Hu W., Fu L., Zhang W., Li L. (2022). Small-Molecule Quenchers for Förster Resonance Energy Transfer: Structure, Mechanism, and Applications. Angew. Chem. Int. Ed. Engl..

[81] Kundu S., Das S., Patra A. (2023). Fluorescence correlation spectroscopy and fluorescence lifetime imaging microscopy for deciphering the morphological evolution of supramolecular self-assembly. Chem. Commun..

[82] Millar D.P. (1996). Time-resolved fluorescence spectroscopy. Curr. Opin. Struct. Biol..

[83] Krichevsky O., Bonnet G. (2002). Fluorescence correlation spectroscopy: The technique and its applications. Rep. Prog. Phys..

[84] Kask P., Palo K., Ullmann D., Gall K. (1999). Fluorescence-intensity distribution analysis and its application in biomolecular detection technology. Proc. Natl. Acad. Sci. USA.

[85] Ning J., Liu T., Dong P., Wang W., Ge G., Wang B., Yu Z., Shi L., Tian X., Huo X. (2019). Molecular Design Strategy to Construct the Near-Infrared Fluorescent Probe for Selectively Sensing Human Cytochrome P450 2J2. J. Am. Chem. Soc..

[86] Tian Z., Yan F., Tian X., Feng L., Cui J., Deng S., Zhang B., Xie T., Huang S., Ma X. (2022). A NIR fluorescent probe for Vanin-1 and its applications in imaging, kidney injury diagnosis, and the development of inhibitor. Acta Pharm. Sin. B.

[87] Zhou H., Tang J., Zhang J., Chen B., Kan J., Zhang W., Zhou J., Ma H. (2019). A red lysosome-targeted fluorescent probe for carboxylesterase detection and bioimaging. J. Mater. Chem. B.

[88] Wu X., Shi W., Li X., Ma H. (2019). Recognition Moieties of Small Molecular Fluorescent Probes for Bioimaging of Enzymes. Acc. Chem. Res..

[89] Zhao J., Chen J., Ma S., Liu Q., Huang L., Chen X., Lou K., Wang W. (2018). Recent developments in multimodality fluorescence imaging probes. Acta Pharm. Sin. B.

[90] Wang P., Yang H., Liu C., Qiu M., Ma X., Mao Z., Sun Y., Liu Z. (2021). Recent advances in the development of activatable multifunctional probes for in vivo imaging of caspase-3. Chin. Chem. Lett..

[91] Saario S.M., Savinainen J.R., Laitinen J.T., Järvinen T., Niemi R. (2004). Monoglyceride lipase-like enzymatic activity is responsible for hydrolysis of 2-arachidonoylglycerol in rat cerebellar membranes. Biochem. Pharmacol..

[92] King A.R., Lodola A., Carmi C., Fu J., Mor M., Piomelli D. (2009). A critical cysteine residue in monoacylglycerol lipase is targeted by a new class of isothiazolinone-based enzyme inhibitors. Br. J. Pharmacol..

[93] Ghafouri N., Tiger G., Razdan R.K., Mahadevan A., Pertwee R.G., Martin B.R., Fowler C.J. (2004). Inhibition of monoacylglycerol lipase and fatty acid amide hydrolase by analogues of 2-arachidonoylglycerol. Br. J. Pharmacol..

[94] Brengdahl J., Fowler C.J. (2006). A novel assay for monoacylglycerol hydrolysis suitable for high-throughput screening. Anal. Biochem..

[95] Muccioli G.G., Labar G., Lambert D.M. (2008). CAY10499, a novel monoglyceride lipase inhibitor evidenced by an expeditious MGL assay. ChemBioChem.

[96] Kanaoka Y., Sekine T., Machida M., Soma Y., Tanizawa K., Ban Y. (1964). Studies on protein-sulfhydryl reagents. I. Synthesis of benzimidazole derivatives of maleimide; fluorescent labeling of maleimide. Chem. Pharm. Bull..

[97] Casida J.E., Gulevich A.G., Sarpong R., Bunnelle E.M. (2010). S-Arachidonoyl-2-thioglycerol synthesis and use for fluorimetric and colorimetric assays of monoacylglycerol lipase. Bioorg. Med. Chem..

[98] Yang J.-R., Langmuir M.E. (1991). Synthesis and properties of a maleimide fluorescent thiol reagent derived from a naphthopyranone. J. Heterocycl. Chem..

[99] Wang Y., Chanda P., Jones P.G., Kennedy J.D. (2008). A fluorescence-based assay for monoacylglycerol lipase compatible with inhibitor screening. Assay Drug Dev. Technol..

[100] Ramarao M.K., Murphy E.A., Shen M.W.H., Wang Y., Bushell K.N., Huang N., Pan N., Williams C., Clark J.D. (2005). A fluorescence-based assay for fatty acid amide hydrolase compatible with high-throughput screening. Anal. Biochem..

[101] Savinainen J.R., Yoshino M., Minkkilä A., Nevalainen T., Laitinen J.T. (2010). Characterization of binding properties of monoglyceride lipase inhibitors by a versatile fluorescence-based technique. Anal. Biochem..

[102] Sun W.C., Gee K.R., Haugland R.P. (1998). Synthesis of novel fluorinated coumarins: Excellent UV-light excitable fluorescent dyes. Bioorg. Med. Chem. Lett..

[103] Clemente J.C., Nulton E., Nelen M., Todd M.J., Maguire D., Schalk-Hihi C., Kuo L.C., Zhang S.-P., Flores C.M., Kranz J.K. (2012). Screening and characterization of human monoglyceride lipase active site inhibitors using orthogonal binding and functional assays. J. Biomol. Screen..

[104] Lauria S., Casati S., Ciuffreda P. (2015). Synthesis and characterization of a new fluorogenic substrate for monoacylglycerol lipase and application to inhibition studies. Anal. Bioanal. Chem..

[105] Miceli M., Casati S., Ottria R., Di Leo S., Eberini I., Palazzolo L., Parravicini C., Ciuffreda P. (2019). Set-Up and Validation of a High Throughput Screening Method for Human Monoacylglycerol Lipase (MAGL) Based on a New Red Fluorescent Probe. Molecules.

[106] Miceli M., Casati S., Allevi P., Berra S., Ottria R., Rota P., Branchini B.R., Ciuffreda P. (2021). A New Ultrasensitive Bioluminescence-Based Method for Assaying Monoacylglycerol Lipase. Int. J. Mol. Sci..

[107] Branchini B.R., Southworth T.L., Fontaine D.M., Kohrt D., Talukder M., Michelini E., Cevenini L., Roda A., Grossel M.J. (2015). An enhanced chimeric firefly luciferase-inspired enzyme for ATP detection and bioluminescence reporter and imaging applications. Anal. Biochem..

[108] Deng H., Zhang Q., Lei Q., Yang N., Yang K., Jiang J., Yu Z. (2022). Discovering monoacylglycerol lipase inhibitors by a combination of fluorogenic substrate assay and activity-based protein profiling. Front. Pharmacol..

[109] Jiang M., Huizenga M.C.W., Wirt J.L., Paloczi J., Amedi A., van den Berg R.J.B.H.N., Benz J., Collin L., Deng H., Di X. (2023). A monoacylglycerol lipase inhibitor showing therapeutic efficacy in mice without central side effects or dependence. Nat. Commun..

[110] Savinainen J.R., Navia-Paldanius D., Laitinen J.T. (2016). A Sensitive and Versatile Fluorescent Activity Assay for ABHD6. Methods Mol. Biol..

[111] Savinainen J.R., Navia-Paldanius D., Laitinen J.T. (2016). A Sensitive and Versatile Fluorescent Activity Assay for ABHD12. Methods Mol. Biol..

[112] Maccarrone M., Bari M., Agrò A.F. (1999). A sensitive and specific radiochromatographic assay of fatty acid amide hydrolase activity. Anal. Biochem..

[113] Omeir R.L., Chin S., Hong Y., Ahern D.G., Deutsch D.G. (1995). Arachidonoyl ethanolamide-1,2-14C as a substrate for anandamide amidase. Life Sci..

[114] Giang D.K., Cravatt B.F. (1997). Molecular characterization of human and mouse fatty acid amide hydrolases. Proc. Natl. Acad. Sci. USA.

[115] Patricelli M.P., Lashuel H.A., Giang D.K., Kelly J.W., Cravatt B.F. (1998). Comparative characterization of a wild type and transmembrane domain-deleted fatty acid amide hydrolase: Identification of the transmembrane domain as a site for oligomerization. Biochemistry.

[116] Thumser A.E., Voysey J., Wilton D.C. (1997). A fluorescence displacement assay for the measurement of arachidonoyl ethanolamide (anandamide) and oleoyl amide (octadecenoamide) hydrolysis. Biochem. Pharmacol..

[117] de Bank P.A., Kendall D.A., Alexander S.P.H. (2005). A spectrophotometric assay for fatty acid amide hydrolase suitable for high-throughput screening. Biochem. Pharmacol..

[118] Wang Y., Ramirez F., Krishnamurthy G., Gilbert A., Kadakia N., Xu J., Kalgaonkar G., Ramarao M.K., Edris W., Rogers K.E. (2006). High-throughput screening for the discovery of inhibitors of fatty acid amide hydrolase using a microsome-based fluorescent assay. J. Biomol. Screen..

[119] Kage K.L., Richardson P.L., Traphagen L., Severin J., Pereda-Lopez A., Lubben T., Davis-Taber R., Vos M.H., Bartley D., Walter K. (2007). A high throughput fluorescent assay for measuring the activity of fatty acid amide hydrolase. J. Neurosci. Methods.

[120] Huang H., Nishi K., Tsai H.-J., Hammock B.D. (2007). Development of highly sensitive fluorescent assays for fatty acid amide hydrolase. Anal. Biochem..

[121] Patricelli M.P., Cravatt B.F. (2001). Characterization and manipulation of the acyl chain selectivity of fatty acid amide hydrolase. Biochemistry.

[122] Dato F.M., Maaßen A., Goldfuß B., Pietsch M. (2018). Characterization of fatty acid amide hydrolase activity by a fluorescence-based assay. Anal. Biochem..

[123] Forster L., Schulze Elfringhoff A., Lehr M. (2009). High-performance liquid chromatographic assay with fluorescence detection for the evaluation of inhibitors against fatty acid amide hydrolase. Anal. Bioanal. Chem..

[124] Tian X., Liu T., Li L., Shao B., Yao D., Feng L., Cui J., James T.D., Ma X. (2020). Visual High-Throughput Screening for Developing a Fatty Acid Amide Hydrolase Natural Inhibitor Based on an Enzyme-Activated Fluorescent Probe. Anal. Chem..

[125] Casasampere M., Ung J., Iñáñez A., Dufau C., Tsuboi K., Casas J., Tan S.-F., Feith D.J., Andrieu-Abadie N., Segui B. (2024). A fluorogenic substrate for the detection of lipid amidases in intact cells. J. Lipid Res..

[126] Sanllehí P., Casasampere M., Abad J.-L., Fabriàs G., López O., Bujons J., Casas J., Delgado A. (2017). The first fluorogenic sensor for sphingosine-1-phosphate lyase activity in intact cells. Chem. Commun..

[127] Tsuboi K., Ueda N. (2023). Assay of NAAA Activity. Methods Mol. Biol..

[128] Solorzano C., Zhu C., Battista N., Astarita G., Lodola A., Rivara S., Mor M., Russo R., Maccarrone M., Antonietti F. (2009). Selective N-acylethanolamine-hydrolyzing acid amidase inhibition reveals a key role for endogenous palmitoylethanolamide in inflammation. Proc. Natl. Acad. Sci. USA.

[129] West J.M., Zvonok N., Whitten K.M., Wood J.T., Makriyannis A. (2012). Mass spectrometric characterization of human N-acylethanolamine-hydrolyzing acid amidase. J. Proteome Res..

[130] Yang L., Ji C., Li Y., Hu F., Zhang F., Zhang H., Li L., Ren J., Wang Z., Qiu Y. (2020). Natural Potent NAAA Inhibitor Atractylodin Counteracts LPS-Induced Microglial Activation. Front. Pharmacol..

[131] West J.M., Zvonok N., Whitten K.M., Vadivel S.K., Bowman A.L., Makriyannis A. (2012). Biochemical and mass spectrometric characterization of human N-acylethanolamine-hydrolyzing acid amidase inhibition. PLoS ONE.

[132] Tian M., Tian Z., Yao D., Ning J., Deng S., Feng L., Huo X., Tian X., Zhang B., Wang C. (2021). A NIR fluorescent probe for fatty acid amide hydrolase bioimaging and its application in development of inhibitors. J. Mater. Chem. B.

[133] Gu X., Wang X., Cai W., Han Y., Zhang Q.-W. (2024). Monofluorophore-based Two-Photon Ratiometric Fluorescent Probe for the Quantitative Imaging of Fatty Acid Amide Hydrolase in Live Neurons and Mouse Brain Tissues. ACS Sens..

[134] Xie Y., Fang Y., Huang Z., Tallon A.M., am Ende C.W., Fox J.M. (2020). Divergent Synthesis of Monosubstituted and Unsymmetrical 3,6-Disubstituted Tetrazines from Carboxylic Ester Precursors. Angew. Chem. Int. Ed. Engl..

[135] Mohammad I., Liebmann K.L., Miller S.C. (2023). Firefly luciferin methyl ester illuminates the activity of multiple serine hydrolases. Chem. Commun..

